# The mTORC1/eIF4E/HIF-1α Pathway Mediates Glycolysis to Support Brain Hypoxia Resistance in the Gansu Zokor, *Eospalax cansus*

**DOI:** 10.3389/fphys.2021.626240

**Published:** 2021-02-23

**Authors:** Jinyan Lin, Lele Fan, Yuming Han, Juanjuan Guo, Zhiqiang Hao, Lingna Cao, Jiamin Kang, Xiaoqin Wang, Jianping He, Jingang Li

**Affiliations:** National Engineering Laboratory for Resource Development of Endangered Crude Drugs in Northwest China, Key Laboratory of the Ministry of Education for Medicinal Resources and Natural Pharmaceutical Chemistry, College of Life Science, Shaanxi Normal University, Xi’an, China

**Keywords:** brain, hypoxia, mTORC1, fructose-driven glycolysis, Gansu zokor (*Eospalax cansus*)

## Abstract

The Gansu zokor (*Eospalax cansus*) is a subterranean rodent species that is unique to China. These creatures inhabit underground burrows with a hypoxia environment. Metabolic energy patterns in subterranean rodents have become a recent focus of research; however, little is known about brain energy metabolism under conditions of hypoxia in this species. The mammalian (mechanistic) target of rapamycin complex 1 (mTORC1) coordinates eukaryotic cell growth and metabolism, and its downstream targets regulate hypoxia inducible factor-1α (HIF-1α) under conditions of hypoxia to induce glycolysis. In this study, we compared the metabolic characteristics of hypoxia-tolerant subterranean Gansu zokors under hypoxic conditions with those of hypoxia-intolerant Sprague-Dawley rats with a similar-sized surface area. We exposed Gansu zokors and rats to hypoxia I (44 h at 10.5% O_2_) or hypoxia II (6 h at 6.5% O_2_) and then measured the transcriptional levels of mTORC1 downstream targets, the transcriptional and translational levels of glycolysis-related genes, glucose and fructose levels in plasma and brain, and the activity of key glycolysis-associated enzymes. Under hypoxia, we found that *hif-1*α transcription was upregulated *via* the mTORC1/eIF4E pathway to drive glycolysis. Furthermore, Gansu zokor brain exhibited enhanced fructose-driven glycolysis under hypoxia through increased expression of the GLUT5 fructose transporter and ketohexokinase (KHK), in addition to increased KHK enzymatic activity, and utilization of fructose; these changes did not occur in rat. However, glucose-driven glycolysis was enhanced in both Gansu zokor and rat under hypoxia II of 6.5% O_2_ for 6 h. Overall, our results indicate that on the basis of glucose as the main metabolic substrate, fructose is used to accelerate the supply of energy in Gansu zokor, which mirrors the metabolic responses to hypoxia in this species.

## Introduction

Although mammals are largely intolerant of hypoxia and their brains exhibit exquisite sensitivity to hypoxia, a few species, such as subterranean rodents, inhabit hypoxic niches and are able to survive hypoxia (Su et al., 2013; Nayak et al., 2016; Grimes et al., 2017; Altwasser et al., 2019; Li et al., 2019; Pamenter et al., 2019; Dong et al., 2020). These subterranean rodents have evolved various strategies to cope with the harsh environmental pressures of the hypoxic environment. All six African mole-rat species have sufficient antioxidant capacity to respond to dynamic hypoxia (Logan et al., 2020), *Lasiopodomys mandarinus*, *Spalax ehrenbergi* (the blind mole rat) and *Heterocephalus glaber* (the naked mole rat) all show hypoxia adaptation as an enhanced capacity for DNA repair and damage prevention (Shams et al., 2013; Lewis et al., 2016; Dong et al., 2020). *Eospalax fontanierii* exhibits reduced blood circulation resistance under hypoxia, which has a protective effect of myocardial function (Xu et al., 2019). These adaptations indicate that subterranean rodents have evolved hypoxic defense mechanisms, which confer protection against a low-oxygen environment.

Hypoxia is an environmental challenge for fossorial species that inhabit underground burrows. One of the greatest challenges in this environment is the high energy cost of burrowing in order to find limited food resources underground and maintain the burrow structure (Vleck, 1979, 1981; Šumbera et al., 2007; Barčiová et al., 2009). Maintaining the balance between energy production and consumption is the key to hypoxia tolerance, which protects against cell death (Staples and Buck, 2009; Speers-Roesch et al., 2010). The mammalian (or mechanistic) target of rapamycin (mTOR), which is a serine/threonine protein kinase in the PI3K-related kinase (PIKK) family, plays a vital role in energy metabolism and cell growth. It exists in two complexes: mTORC1 and mTORC2 (Saxton and Sabatini, 2017; Yan et al., 2017). mTORC1 controls the balance of metabolism in response to hypoxic conditions. The mTORC1/eIF4E pathway regulates hypoxia inducible factor 1α (HIF-1α), which plays an important role in glycolysis (Cheng et al., 2016; Chi et al., 2017; Saxton and Sabatini, 2017). HIF-1α stimulates the expression of transporters, glycolytic enzymes and glycolytic-inducing factors to regulate glycolysis (Zhang and Sadek, 2014). Glucose and fructose enter cells *via* the GLUT1 and GLUT5 transporters, respectively. GLUT5 is a highly selective transporter for fructose (Inukai et al., 1995). In the glucose-driven glycolysis pathway, phosphofructokinase (PFK) catalyzes the rate-limiting step from fructose-6-phosphate to fructose-1,6-bisphosphate (Real-Hohn et al., 2010). In the fructose-driven glycolysis pathway, ketohexokinase (KHK) catalyzes the direct phosphorylation of fructose to form fructose 1-phosphate, thus bypassing the PFK regulatory block and allowing continued glycolytic flux independent of cellular energy status (Park et al., 2017).

There have been some studies on the mechanisms underlying adaptive responses to hypoxia in subterranean rodents in recent years (Park et al., 2017; Schmidt et al., 2017; Faulkes et al., 2019; Pamenter et al., 2019; Farhat et al., 2020), but these have been mainly limited to naked mole rats and blind mole rats and further studies on other subterranean rodent species are needed. The family Spalacidae includes a group of naturally subterranean rodents known as zokors. Zokors (*Eospalax*) spend 85–90% of their time in self-constructed underground burrows, rarely venturing out on the ground surface; on the one hand, the burrows offer shelter from extreme climatic conditions and predators, while on the other hand, zokors must withstand the stress of a hypoxic and hypercapnic environment in almost total darkness burrows (Zhou and Dou, 1990; Zhang et al., 2003; Lacey et al., 2010; Xu et al., 2019). Furthermore, these animals require large amounts of energy digging to locate unpredictably and unevenly scattered food resources (Begall et al., 2007). Zokors have a marked influence on the soil texture and water-holding capacity by excavating vast burrow systems, which play an important role in the ecosystem structure and development (Zhang et al., 2003). The Gansu zokor (*Eospalax cansus*) is unique to China and endemic to the Loess plateau, inhabiting prairie, forest, meadow and farmland habitats (Su et al., 2014; Xu et al., 2019). The foraging activity of Gansu zokor is conducted mainly at a depth of 8–13 cm under the ground, and they rest in nest at the bottom of the burrow, which are usually 1–2 m deep. Their burrow systems are approximately 10 cm in diameter, with extending to approximately 100 m (Chen and Huang, 2012; Pleštilová et al., 2018). During the recent decades, studies have been conducted on the population age of Gansu zokor (Li and Wang, 1992) and seismic communication (Li et al., 2001), as well as harms and their prevention (Yang and Wang, 1992; Wang, 2010). Recently, hypoxia adaptation in Gansu zokor has received increasing attention to provide a theoretical basis for hypoxic injury treatment (Tang et al., 2013; Shan et al., 2016; Zhang et al., 2016; Meng et al., 2017). In the laboratory, Gansu zokor tolerates 3% O_2_ for more than 1 h and 4% O_2_ for more than 10 h. As a hypoxia-intolerant species, rats survive for only 16 min at 3% O_2_ and 4 h at 4% O_2_ (Yan et al., 2012). Moreover, studies have shown that Gansu zokor have higher red blood cell (RBC) counts, hemoglobin concentration (HGB), hematocrit (HCT), and vessel density (Xie et al., 2012; Shan et al., 2016) as well as higher cardiac activities of superoxide dismutase (SOD), catalase (CAT), and glutathione reductase (GR) compared with rats under hypoxic conditions (Tang et al., 2013). These findings indicate that Gansu zokor maintains hypoxia tolerance through increased oxygen-carrying capacity and antioxidant enzyme activity. The hypoxia tolerance capacity of Gansu zokor may be the result of adaptations to the putatively mild hypoxic environment in their sealed burrows. Furthermore, these rodents probably experience several hours of severe hypoxia in their deep nests during periods of extreme weather, such as rainy season.

**FIGURE 1:**
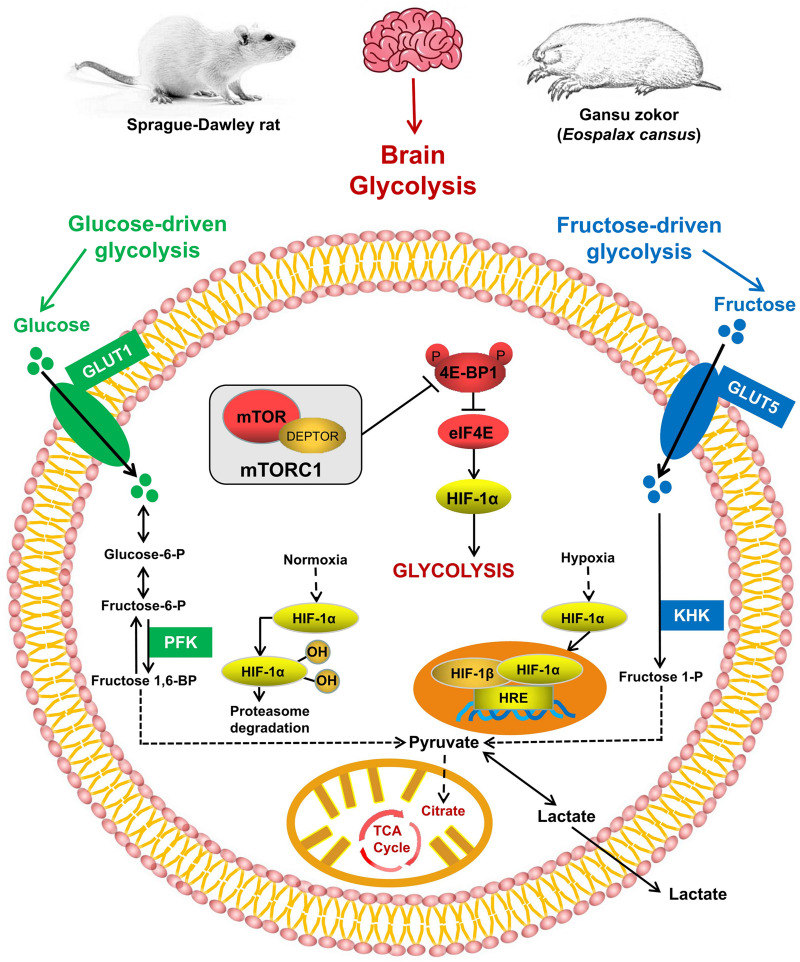
The mTORC1 signaling and glycolysis pathways. mTORC1 controls the activity of several transcription factors (shown in red) that drive HIF-1α expression. Glucose enters the brain *via* GLUT1 and is converted *via* phosphofructokinase (PFK; shown in green); fructose enters cells *via* GLUT5 and is phosphorylated by ketohexokinase (KHK; shown in blue).

Due to its almost completely aerobic metabolism, the brain is the most hypoxia-sensitive organ and requires a higher oxygen consumption and energy demand than other tissues (Buck and Pamenter, 2018; Sun et al., 2018; Logan et al., 2020). Studies showed that the naked mole rat brain has evolved the ability to alter glycolytic substrates using fructose in near-anaerobic metabolism, which supports the viability of this species under conditions of oxygen deprivation (Park et al., 2017). Furthermore, energy metabolism is suppressed and the lipid composition of membranes is altered in the naked mole rat brain in response to moderate hypoxia (Farhat et al., 2020). These findings indicate that metabolic “rewiring” may be a mechanism by which hypoxic brain damage is minimized. However, little is known about the underlying mechanism of brain energy metabolism in subterranean rodents *in vivo* under the hypoxic conditions that might occur in subterranean burrows. Gansu zokor represents an ideal natural model of subterranean rodents that can be used to study the mechanism of hypoxia tolerance. Here we hypothesize that under the hypoxic condition of subterranean burrow, Gansu zokor could also mediate the rewired glycolysis to support its brain hypoxia resistance. Thus, we focused on brain glycolysis mediated *via* the mTORC1/eIF4E/HIF-1α pathway under hypoxia (Figure 1) and investigated the mechanisms by which Gansu zokor cope with these conditions. We addressed these issues by performing an *in vivo* comparison of the subterranean hypoxia-tolerant Gansu zokor and the surface hypoxia-intolerant Sprague-Dawley rat under normoxic (21% O_2_) and hypoxic (10.5% O_2_ or 6.5% O_2_) conditions. The hypoxic conditions used reflect the relatively mild hypoxia to which Gansu zokor might be exposed while rebuilding collapsed burrows (10.5% O_2_), and the relative acute hypoxia that might occur at the bottom of the subterranean burrow during the rainy season (6.5% O_2_) (Shams et al., 2005; Band et al., 2010; Moskovitz et al., 2012). Whole brain RNA and protein were extracted to detect the transcriptional levels of mTORC1/eIF4E/HIF-1α pathway genes, and the transcriptional and translational levels of glycolysis-related genes. We then tested the activity of key enzymes involved in glucose-driven and fructose-driven glycolysis, with the aim of revealing the molecular mechanisms involved in energy supply in subterranean rodents under hypoxic conditions.

## Materials and Methods

### Animals and Hypoxia Treatment

**FIGURE 2:**
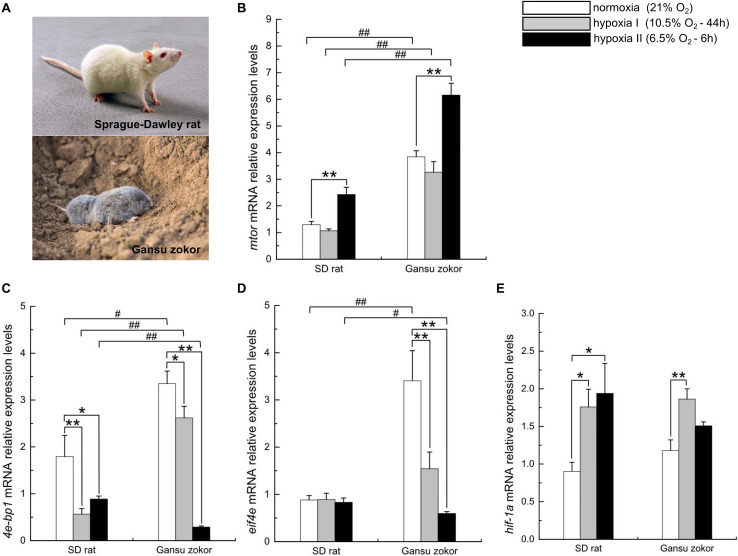
Transcription level analysis of the mTORC1/eIF4E/HIF-1α pathway in Sprague-Dawley rat [**(A)**, top; this image from the network: https://baike.baidu.com/item/%E5%A4%A7%E7%99%BD%E9%BC%A0/6868090?fr=aladdin] and Gansu zokor [**(A)**, bottom; this image from the network: https://news.qq.com/a/20150330/064242.htm?pc#p=2]. *mtor* **(B)**, *4e-bp1* **(C)**, *eif4e* **(D)**, and *hif-1*α **(E)** mRNA expression. All data are presented as mean ± SEM (*n* = 6 animals per species). Statistical symbols: #*P* < 0.05 and ##*P* < 0.01 between two species under the same oxygen treatments; *P < 0.05 and **P < 0.01 between different oxygen treatments in the same species.

Gansu zokors (Figure 2A, bottom) were captured from the wild during the month of June in Weinan, Shaanxi Province, China (35°4′N, 109°16′E). Animals were housed in individual cages (47.5 cm × 35.0 cm × 20.0 cm) at room temperature under a 12:12 h light-dark cycle and acclimated to laboratory conditions for 3 weeks prior to exposure to hypoxia. Sprague-Dawley rats (rats; Figure 2A, top) were obtained from the Xi’an Jiaotong University and group-housed (three rats per cage) under the same environmental conditions. All animals were fed *ad libitum* (Gansu zokors, carrots; rats, standard rat feed and water).

To mimic hypoxia, nine healthy adult male Gansu zokors (230 ± 25 g) and nine healthy adult female Gansu zokors (210 ± 25 g) were randomly divided into three groups (*n* = 6 animals per species per group; three male and three female): (1) normoxia group (21% O_2_); (2) hypoxia I group (44 h at 10.5% O_2_) and (3) hypoxia II group (6 h at 6.5% O_2_). A hypoxic cabin was used to simulate hypoxia, and an O_2_ analyzer (Junfang, Beijing, China) was used to measure and maintain the O_2_ level at 6.5% O_2_/10.5% O_2_. The O_2_ level was maintained by delivering N_2_ and two plates containing soda-lime were placed in the chamber to absorb the CO_2_ released by the animals. Eighteen healthy adult rats (nine male and nine female; 200 ± 15 g) were treated in the same groups under the same experimental conditions. All experimental animals were sacrificed by injection with pentobarbital sodium at a dose of 45 mg/kg of body weigh. Cardiac blood samples were drawn from the right ventricle, and heparin-plasma was collected after centrifugation (3,000 rpm, 10 min, 4°C). Brain samples were removed and immediately frozen in liquid nitrogen. Plasma and brain samples were stored at −80°C until analyzed.

### RNA Extraction and Quantitative Real-Time PCR Verification

Total RNA was extracted from brain tissues using TRIzol reagent (TaKaRa, Beijing, China) according to the manufacturer’s instructions. The RNA samples were then reverse transcribed to cDNA using a Prime Script II 1st Strand cDNA Synthesis kit (TaKaRa, Beijing, China). Primers were designed using Primer-BLAST, and the sequences are shown in Table 1. Six technical replicates were prepared for the analysis of each gene by quantitative real-time PCR verification (qRT-PCR) using a Step One Real-Time System (ABI). The samples were analyzed in a 25 μl reaction volume, which consisted of 12.5 μl SYBR^®^ Premix Ex TaqII (Tli RNaseH Plus) (2×), 1 μl each primer (10 μM), 8.5 μl nuclease-free water, and 2 μl template cDNA (diluted 30 times). The qRT-PCR conditions were as follows: 95°C for 30 s, followed by 39 cycles of 95°C for 15 s and 60°C for 30 s. Relative gene expression levels were normalized to that of an internal reference gene (β*-actin*) and calculated according to the 2^–ΔΔ*Ct*^ method.

**TABLE 1:** Primers for qRT-PCR analysis.

Gene	Primer	Primer sequence (5′–3′)	Tm (°C)
*Mtor Eospalax cansus*	Forward	CCTCTCTCACCATCACACCA	64
	Reverse	GGCTCTTCACAAAGGACACC	
*Mtor* Sprague- Dawley rat	Forward	GAAGTGAAGCGAGCCTTGGA	62
	Reverse	AATCAGACAGGCACGAAGGG	
*4e-bp1 Eospalax cansus*	Forward	TGAGCCTCCCATGAAAACCA	62
	Reverse	TGCTATGGCTCTCCTCCCAA	
*4e-bp1* Sprague- Dawley rat	Forward	ATTCCTGATGGAGTGTCGGAA	60
	Reverse	CCACCTGCCCGCTTATCTT	
*Eif4e Eospalax cansus*	Forward	GGGAGCAGTTCATGGTTGG	60
	Reverse	TGGTGTTGGGAGGTAGGTTA	
*Eif4e* Sprague- Dawley rat	Forward	GGGAGCAGTTCATGGTTGG	60
	Reverse	TGGTGTTGGGAGGTAGGTTA	
*hif-1*α *Eospalax cansus*	Forward	CTCATTTTGCCGCAGTGCCT	62
	Reverse	CGCACCATTCCTCGCCATAA	
*Hif-1*α Sprague- Dawley rat	Forward	CCAGATTCAAGATCAGCCAGCA	62
	Reverse	GCTGTCCACATCAAAGCAGTACTCA	
*glut1 Eospalax cansus*	Forward	ATTGTGCTGCTGACCTTACC	58
	Reverse	GTTCCTAAATGGATGGAGCCTA	
*glut1* Sprague- Dawley rat	Forward	ATTGTGCTGCTGACCTTACC	58
	Reverse	GTTCCTAAATGGATGGAGCCTA	
*Pfk Eospalax cansus*	Forward	CGACCGTATCTTGAGCAGCAA	62
	Reverse	CGTCAAACCTCTTGTCATCCA	
*Pfk* Sprague- Dawley rat	Forward	CGACCGTATCTTGAGCAGCAA	62
	Reverse	CGTCAAACCTCTTGTCATCCA	
*glut5 Eospalax cansus*	Forward	AATCCGGAAGGAGGATGAGG	61
	Reverse	TGCGCTGAGGTAGATCTGGT	
*glut5* Sprague- Dawley rat	Forward	AATCCGGAAGGAGGATGAGG	61
	Reverse	TGCGCTGAGGTAGATCTGGT	
*Khk Eospalax cansus*	Forward	AAGCAGATCCTGTGCGTGG	62
	Reverse	CCAGCGAGCCCATGAAGG	
*Khk* Sprague- Dawley rat	Forward	AAGCAGATCCTGTGCGTGG	62
	Reverse	CCAGCGAGCCCATGAAGG	
β*-actin*	Forward	CTAAGGCCAACCGTGAAAAGAT	60 ± 5
	Reverse	GACCAGAGGCATACAGGGACA	

### Western Blot Analysis

Brain samples were homogenized and transferred to a separate tube containing RIPA buffer for 35 min on ice. After centrifugation (13,000 rpm, 30 min, 4°C), protein concentrations were measured in the resulting supernatants using a BCA kit (Coolaber, Beijing, China). Protein lysates (20 μg per well) were separated by sodium dodecyl sulfate-polyacrylamide gel (10%) electrophoresis (SDS-PAGE) and transferred to polyvinylidene fluoride (PVDF) membranes. After blocking in 5% skimmed milk for 2 h at room temperature on a shaking platform, the membranes were incubated overnight at 4°C with primary antibodies for the detection of GLUT1 (1:8,000; Abcam, Cambridge, MA, United States), PFK (1:1,000; Abcam, Cambridge, MA, United States), GLUT5 (1:500; Affinity Biosciences, Cincinnati, OH, United States), KHK (1:2,500; Abcam, Cambridge, MA, United States) and β-actin (1:8,000; ABclonal, Wuhan, China). The membranes were then washed and incubated with the secondary detection antibody (1:3,000; horseradish peroxidase-conjugated anti-rabbit IgG; ABclonal, Wuhan, China). Immunoreactive protein bands were visualized using an ECL Western blot substrate (Pierce, Rockford, IL, United States) and images were captured on an Alpha Innotech Chemilmager (Alpha Innotech, San Leandro, CA, United States). Quantification of the proteins was carried out using Image J Software.

### Immunohistochemical Staining

Brain sections were incubated in 3% H_2_O_2_ for 20 min to quench endogenous peroxidase activity. Then sections were incubated with 5% goat serum to block non-specific staining. The sections were incubated overnight at 4°C with primary antibodies for the detection of GLUT1 (1:500; Abcam, Cambridge, MA, United States), PFK (1:50; Abcam, Cambridge, MA, United States), GLUT5 (1:100; Affinity Biosciences, Cincinnati, OH, United States), and KHK (1:500; Abcam, Cambridge, MA, United States). The sections were then washed and incubated with horseradish peroxidase (Maixin, Fuzhou, China) for 50 min at room temperature. Signals were visualized using the diaminobenzidine (DAB) chromogenic kit. The negative control was treated with PBS in place of primary antibodies. Then sections were counterstained with hematoxylin and were viewed by optical microscopy. The Image Pro Plus 6.0 software was applied to determine the integral optical density (IOD) values of proteins.

### Enzymatic Activity Assay

Brain samples were placed at room temperature for 1 h after storage at 4°C for 12 h. PBS (10 times the volume of brain tissue) was then added for homogenization. The supernatant samples were collected after centrifugation (5,000 rpm, 15 min, room temperature) and the activity of PFK and KHK enzymes was measured immediately using enzyme-linked immunoassay (ELISA) kits (PFK kit, Kete, Yancheng, China and KHK kit, Meimian, Yancheng, China), according to the manufacturers’ instructions, with six biological replicates per treatment.

### Glucose and Fructose Determination

Approximately 0.1 g brain tissue was placed into 1 ml dd H_2_O, homogenized and centrifuged to collect the supernatant (8,000 × *g*, 10 min, room temperature). The glucose and fructose contents in supernatant samples and plasma were assessed using a glucose content kit (Feiya, Yancheng, China) and a fructose content kit (Feiya, Yancheng, China) according to the manufacturers’ instructions.

### Statistical Analysis

Data were presented as mean values ± standard error of mean (SEM). Differences between two species at the same oxygen concentration were evaluated using independent sample *t-*tests, and differences within the same species at different oxygen concentrations were evaluated using *post hoc* tests. *P*-values < 0.05 were considered to indicate statistical significance.

## Results

### Quantitative Analysis of the Expression of Genes Related to the mTORC1/eIF4E/HIF-1α Pathway

We first analyzed the expression levels of the *mtor*, *4e-bp1*, *eif4e* and *hif-1*α genes in Gansu zokor under conditions of hypoxia compared with those under normoxia by qRT-PCR using the primers shown in Table 1. We observed that the expression of *mtor* mRNA was significantly increased in the hypoxia II group (*P* < 0.01) (Figure 2B), while the expression levels of *4e-bp1* and *eif4e*, which are downstream genes in the mTORC1 pathway, decreased gradually with as the oxygen concentration was reduced (Figures 2C,D). Expression of *hif-1*α mRNA was significantly increased in the hypoxia I group (*P* < 0.01), with a slight increase in the hypoxia II group (Figure 2E).

We then made a major observation by comparing the expression of these genes expression in Gansu zokor and the hypoxia-intolerant rat; the expression levels of *mtor* mRNA in Gansu zokor were significantly higher than those in rat under conditions of normoxia as well as the two hypoxia conditions (*P* < 0.01) (Figure 2B). Compared with rats, the expression levels of *4e-bp1* and *eif4e* mRNA in Gansu zokors were significantly higher in the normoxia group and significantly lower in the hypoxia II group (Figures 2C,D). Furthermore, there were no significant differences in *hif-1*α mRNA expression between the two species in all groups (Figure 2E).

### Quantitative Analysis of Glycolysis-Related Gene Expression

**FIGURE 3:**
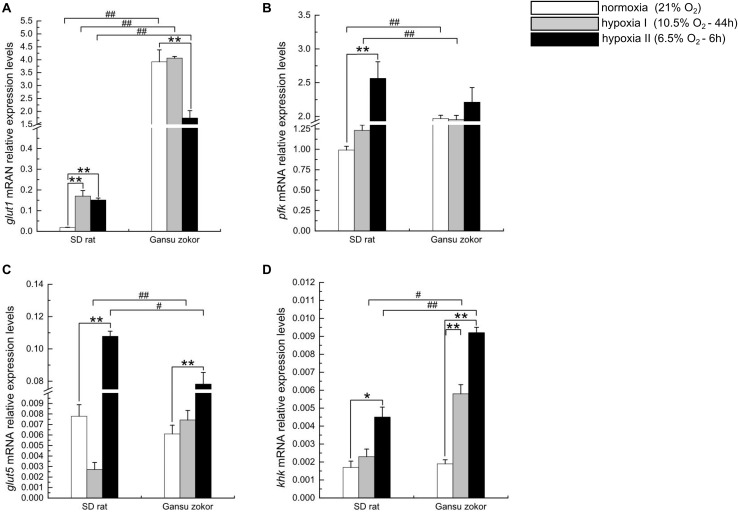
Transcription level analysis of glycolysis-related transporters and enzymes in Sprague-Dawley rat and Gansu zokor. *glut1* glucose transporter **(A)**, *pfk* **(B)**, *glut5* glucose transporter **(C)**, and *khk* **(D)** mRNA expression. All data are presented as mean ± SEM (*n* = 6 animals per species). Statistical symbols: #*P* < 0.05 and ##*P* < 0.01 between two species under the same oxygen treatment; *P < 0.05 and **P < 0.01 between different oxygen treatments in the same species.

**FIGURE 4:**
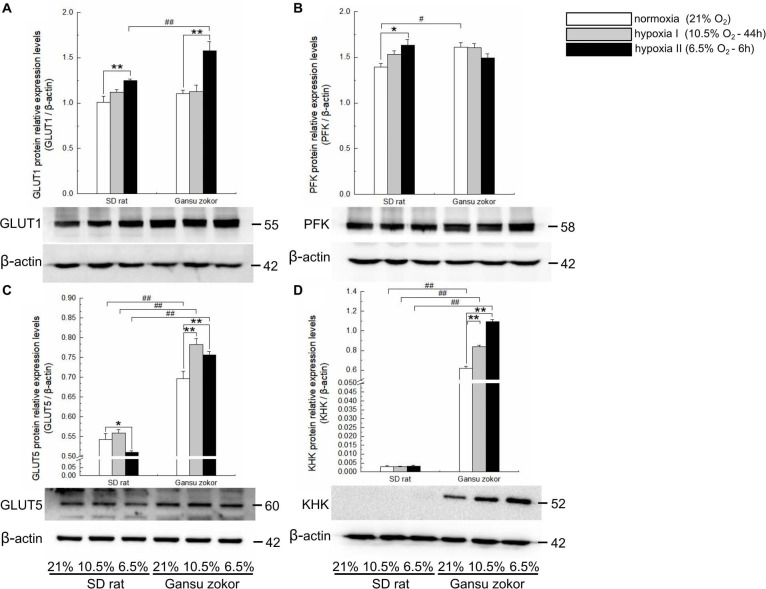
Translation level analysis of glycolysis-related transporters and enzymes in Sprague-Dawley rat and Gansu zokor. GLUT1 glucose transporter **(A)**, PFK **(B)**, GLUT5 glucose transporter **(C)**, and KHK **(D)** protein expression. Representative images of at least three separate blots below each bar graph showing expression of the proteins. All data are presented as mean ± SEM. Statistical symbols: #*P* < 0.05 and ##*P* < 0.01 between two species under the same oxygen treatment; *P < 0.05 and **P < 0.01 between different oxygen treatments in the same species.

We first analyzed the expression levels of *glut1* and *pfk* (glucose-driven glycolysis pathway) and *glut5* and *khk* (fructose-driven glycolysis pathway) in Gansu zokor under conditions of hypoxia compared with those under normoxia by qRT-PCR using the primers shown in Table 1. In the glucose-driven glycolysis pathway, hypoxia II significantly decreased *glut1* mRNA expression (Figure 3A), whereas GLUT1 protein expression was significantly increased (Figure 4A). Hypoxia did not significantly change the *pfk* expression at either the mRNA or protein levels (Figures 3B, 4B). In the fructose-driven glycolysis pathway, both hypoxia I and hypoxia II induced increases in the expression of *glut5* and *khk* mRNA in Gansu zokor (Figures 3C,D), with the protein levels corresponding with the mRNA levels (Figures 4C,D).

The mRNA levels of these glycolysis-related genes were then compared between Gansu zokor and rat and the results revealed differences between the two species. In the glucose-driven glycolysis pathway, *glut1* mRNA expression in Gansu zokor was significantly higher than that in rat both under normoxia and the two hypoxia conditions (*P* < 0.01) (Figure 3A). However, at the protein expression level, this differential expression was found only in the hypoxia II group (Figure 4A). The *pfk* mRNA expression in Gansu zokor was significantly higher than that in rat both under normoxia and hypoxia I conditions (*P* < 0.01) (Figure 3B), but this differential in PFK protein expression was found only under normoxic condition (Figure 4B). In the fructose-driven glycolysis pathway, although the *glut5* and *khk* mRNA expression levels were significantly higher in Gansu zokor than those in rat only in some groups (Figures 3C,D), the GLUT5 and KHK protein expression levels in Gansu zokor were significantly higher than those in rat in all groups (*P* < 0.01) (Figures 4C,D), showing a high degree of consistency. Surprisingly, KHK protein was almost totally absent in rat brain tissue; however, like the PFK protein in the glucose-driven glycolysis pathway, this protein was expressed normally in Gansu zokor, at levels hundreds of times higher than that in rat (Figure 4D), this was an important and interesting finding in our study.

**FIGURE 5:**
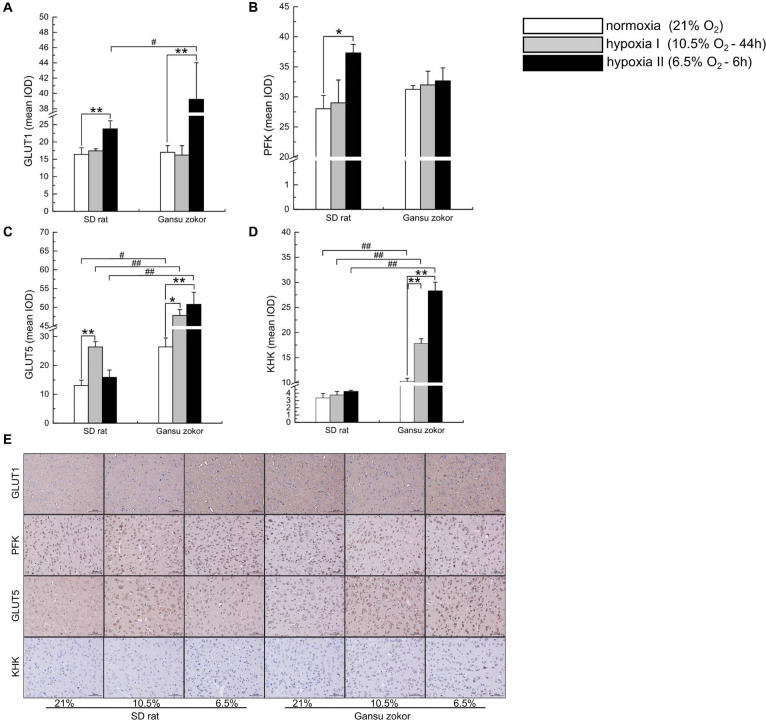
Immunohistochemical analysis of glycolysis-related transporters and enzymes in Sprague-Dawley rat and Gansu zokor. GLUT1 glucose transporter **(A)**, PFK **(B)**, GLUT5 glucose transporter **(C)**, and KHK **(D)** protein immunoreactivity. Representative images **(E)** of immunostaining, scale bar represents: 100 μm, magnification: 200×. All data are presented as mean ± SEM. Statistical symbols: #*P* < 0.05 and ##*P* < 0.01 between two species under the same oxygen treatment; *P < 0.05 and **P < 0.01 between different oxygen treatments in the same species.

We further verified the glycolysis-related protein expression by immunohistochemistry staining (Figure 5E). We analyzed the protein immunoreactivity of GLUT1, PFK, GLUT5, and KHK in Gansu zokor under conditions of hypoxia compared with those under normoxia. In the glucose-driven glycolysis pathway, only the hypoxia II significantly increased GLUT1 protein immunoreactivity (*P* < 0.01) (Figure 5A), and hypoxia did not significantly change the PFK protein immunoreactivity (Figure 5B). In the fructose-driven glycolysis pathway, both hypoxia I and hypoxia II significantly increased GLUT5 and KHK protein immunoreactivity (Figures 5C,D). Furthermore, we also found that the GLUT5 and KHK protein immunoreactivity in Gansu zokor was significantly higher than those in rat in all groups (*P* < 0.01) (Figures 5C,D). These results were consistent with those detected by the Western blot method.

### Quantitative Analysis of Glycolysis-Related Enzymatic Activity

**FIGURE 6:**
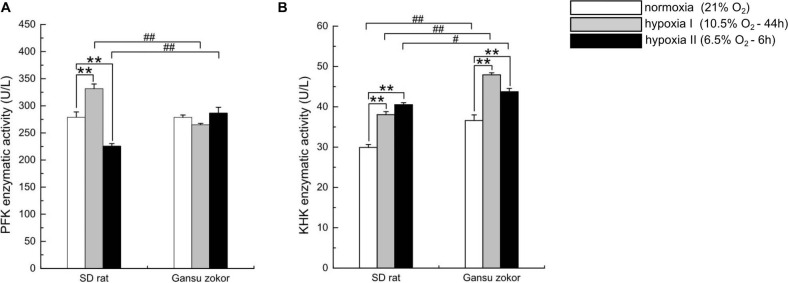
Enzymatic activity analysis of glycolysis in Sprague-Dawley rat and Gansu zokor. The activity of PFK (glucose enzyme) **(A)** and KHK (fructose enzyme) **(B)**. All data are presented as mean ± SEM (*n* = 6 animals per species). Statistical symbols: #*P* < 0.05 and ##*P* < 0.01 between two species under the same oxygen treatment; **P < 0.01 between different oxygen treatments in the same species.

We first observed that hypoxia exposure did not significantly change the enzymatic activity of PFK in Gansu zokor brain (Figure 6A), whereas KHK enzymatic activity was significantly increased (Figure 6B). Then compared with rat, PFK enzymatic activity in Gansu zokor was significantly lower than that in rat in hypoxia I group, whereas the activity was significantly higher in hypoxia II group (Figure 6A). In contrast, KHK enzymatic activity in Gansu zokor was significantly higher in all groups than that in rat (Figure 6B). Thus, we observed differences in the activity of glycolysis-related of Gansu zokor compared with hypoxia-intolerant rats under both normoxic and hypoxic conditions.

### Hypoxia Affects Glucose and Fructose Levels

**FIGURE 7:**
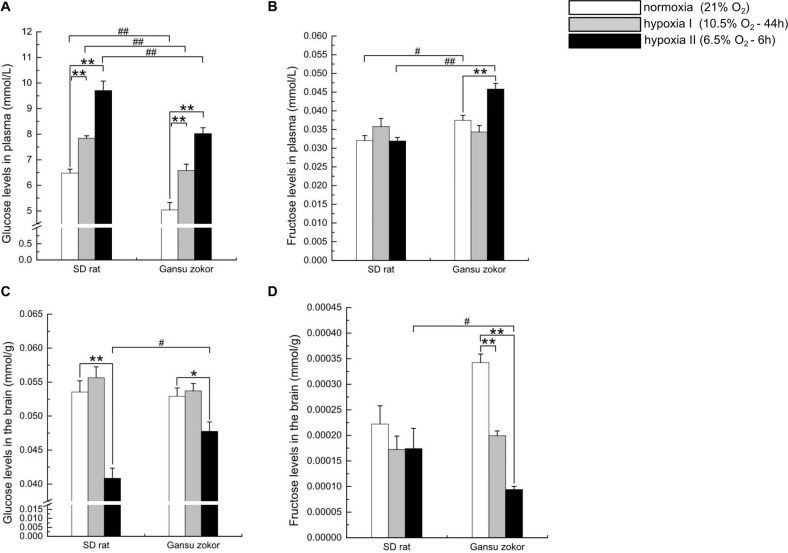
Glucose and fructose levels in the plasma and brain in Sprague-Dawley rat and Gansu zokor. Plasma glucose **(A)**, plasma fructose **(B)**, brain glucose **(C)**, and brain fructose **(D)** levels. All data are presented as mean ± SEM (*n* = 6 animals per species). Statistical symbols: #*P* < 0.05 and ##*P* < 0.01 between two species under the same oxygen treatment; *P < 0.05 and **P < 0.01 between different oxygen treatments in the same species.

To evaluate whether hypoxia affects glucose or fructose levels, we detected the levels of these sugars in plasma and brain. Surprisingly, the plasma glucose levels in Gansu zokor were significantly lower than those in rat both at normoxia and under the two hypoxia conditions (*P* < 0.01) (Figure 7A). In contrast, the plasma fructose levels in Gansu zokor were significantly higher than those in rat under normoxia (*P* < 0.05) and hypoxia II conditions (*P* < 0.01) (Figure 7B). Furthermore, unlike rats, Gansu zokors exhibited raised plasma levels of both glucose and fructose under hypoxia II condition (Figures 7A,B).

Interestingly, although the plasma glucose levels in Gansu zokor were significantly lower than those in rat, there were no significant differences in brain glucose levels between the two species under normoxia and hypoxia I conditions (Figure 7C). Furthermore, rats showed unchanged brain fructose levels after hypoxic exposure (Figure 7D), although Gansu zokors showed significant decreases in two hypoxia groups when compared with normoxia (Figure 7D).

## Discussion

In humans, hypoxia leads to loss of consciousness within minutes. Furthermore, hypoxic-ischemic brain injury has devastating impacts, especially in patients who are comatose after resuscitation from cardiac arrest (Weil et al., 2008; Avivi et al., 2010; Sandroni et al., 2018). However, some subterranean rodent species can tolerate the ambient hypoxia in their underground burrows for prolonged periods (Band et al., 2010; Pamenter et al., 2019). The native subterranean rodent species used in current hypoxia studies have some limitations, and are mainly limited in naked mole rats and blind mole rats. The molecular mechanisms underlying the brain adaptations of subterranean rodents to hypoxic condition, especially in the Gansu zokor, remain to be elucidated. In this study, we investigated the mechanisms underlying brain glycolysis in Gansu zokor under hypoxic conditions by revealing the regulatory effects of the mTORC1/eIF4E/HIF-1α pathway on glycolysis. Our study yielded the following important findings: (i) As a downstream transcription factor in the mTORC1/eIF4E pathway, *hif-1*α gene expression in the brain of both Gansu zokor and rat under hypoxic conditions was increased when compared with normoxia. (ii) Expression of the GLUT5 transmembrane protein, which is a highly selective fructose transporter (Park et al., 2017), was significantly higher in Gansu zokor than that in rat in all the groups. (iii) Under the two different degrees of hypoxia, expression of *khk* in Gansu zokor brain was significantly higher than in rat at both the mRNA and its protein levels. In particular, KHK protein expression was extremely significantly higher in Gansu zokor than that in rat in all the groups. Furthermore, in Gansu zokor, but not in rat, KHK protein expression was upregulated in parallel with the decrease in oxygen concentration. Finally, in terms of enzymatic activity, KHK activity was significantly higher in Gansu zokor than that in rat in all the groups. (*iv*) On the whole, in Gansu zokor, hypoxia exposure significantly increased blood fructose levels, while brain fructose levels were significantly decreased; however, no significant changes in blood or brain fructose levels were observed in rats exposed to hypoxia stress. Based on these findings, we postulated that Gansu zokor have a glycometabolism pattern in response to hypoxia that differs from that of hypoxia-intolerant animals.

The mTOR controls cellular growth and energy (Hall, 2008; Saxton and Sabatini, 2017). In this study, we found significantly higher levels of *mtor* mRNA in Gansu zokor compared with those in rat under all treatment conditions, indicating that Gansu zokors have a high capacity for energy metabolism or/and growth compared with the surface dwelling rat. HIF-1α is known to regulate the expression of downstream genes with roles in the process of glycolysis (Shams et al., 2004; Zhang and Sadek, 2014). Our data support the conclusions of previous studies, showing that mTORC1 signaling promotes *hif-1*α transcription (Dodd et al., 2015; Chi et al., 2017). To gain a better understanding of this process in subterranean rodents *in vivo* under conditions of hypoxia, we designed this study to explore mTORC1-mediated metabolism under hypoxia.

### Fructose-Driven Glycolysis Capability Is Reinforced in Hypoxic Gansu Zokor Brain

Fructolysis occurs primarily in the liver, small intestine, and kidney (Mirtschink et al., 2018); in part because GLUT5, a highly selective fructose transporter, and KHK, which catalyzed the first step in fructose metabolism, are primarily expressed in these organs (Funari et al., 2005; Diggle et al., 2009; Gonzalez and Betts, 2018). However, in an important study, Park et al. showed that naked mole rats have the ability to use fructose to fuel the brain under near anaerobic conditions (Park et al., 2017). This led us to suspect that this fructose metabolism pattern exists in other subterranean rodents living in burrows under conditions of hypoxia, even if this environment is not as extreme as near-anaerobic conditions. Therefore, we exposed Gansu zokors to hypoxic conditions that might occur in underground habitats (Shams et al., 2005). Similar to naked mole rats (Park et al., 2017), we found that Gansu zokor brain showed increased fructose-driven glycolysis in response to hypoxia stress.

GLUT5 expression in Gansu zokor brains is higher than that in the surface-dwelling rat, which is consistent with the previous report in naked mole rat brain (Park et al., 2017). It is also worth noting that under hypoxia II condition, Gansu zokor has lower *glut5* mRNA but higher protein expression than that in rat, which suggests that substrates for fructose-driven glycolysis are rapidly transported in Gansu zokor by improving the translation efficiency of *glut5* mRNA. This mechanism clearly facilitates the rapid activation of the fructose metabolism pathway in Gansu zokor under hypoxia. Furthermore, hypoxia increased GLUT5 protein levels in Gansu zokor brain, which is similar to the stimulation observed in rat rostral ventrolateral medulla (RVLM) in response to a high fructose diet (Wu et al., 2014). This seems to suggest that hypoxia in Gansu zokor produces similar results to that caused by high fructose intake in rat. We then found that plasma fructose is increased to statistically significant levels in the Gansu zokor during hypoxia II condition. Although the source of this fructose is not clear, it is probably produced by endogenous synthesis (Patel et al., 2015). On the other hand, KHK initiates fructose-driven glycolysis to bypass the PFK regulatory block in glucose-driven glycolysis (Diggle et al., 2009; Park et al., 2017). In this study, we showed that KHK protein is expressed at negligible levels in the rat brain, but can be expressed in the Gansu zokor brain. Furthermore, KHK activity in Gansu zokor was significantly higher than that in rat, and was upregulated under hypoxia; consistent results occurred in the expression of the enzyme at both the mRNA and protein levels. These results indicated that Gansu zokor has the capacity to initiate fructose metabolism, especially under conditions of hypoxia; which meant that the fructose-fueled glycolysis pattern occurred in Gansu zokor. Thus, experimental results confirmed our initial hypothesis that Gansu zokor could mediate the rewired glycolysis. This rewired fructose metabolism pathway bypasses metabolic block at PFK, it involves equipping brain with fructose transporter and fructose enzyme for fructose metabolism. Besides, we observed marked differences in the fructose-driven glycolysis pathway between Gansu zokor and rat, suggesting that Gansu zokor has different metabolic patterns from those of the surface-dwelling rat. Fructose enters the glycolysis pathway at a much higher rate than glucose (Park et al., 2017; Liu et al., 2020). The rapid metabolism of fructose is critical for maintaining normal brain metabolism activity in Gansu zokor under hypoxia. This metabolic pattern can circumvent the lethal effects of hypoxia, thus providing a mechanism for survival in the subterranean burrow. Our findings suggested that Gansu zokor brain could adapt to subterranean hypoxia through rewiring the metabolism of glycolysis, which may be attributed to the long-term evolution of Gansu zokor inhabiting hypoxic environments. The adaptive evolution of subterranean rodents is both degenerative and compensatory, thus providing a model of the nature of adaptation through modifications at the molecular and organism levels (Nevo, 1999; Begall et al., 2007). The unique insights in to the glycolysis characteristics in Gansu zokor obtained in our study will be useful for further studies on evolution in subterranean rodents.

### Glycolysis Fuel in Gansu Zokor Brain Strengthen Reliance on Glucose and Fructose Simultaneously Under Hypoxia II Condition

As described previously, Gansu zokors may rely upon fructose-driven glycolysis under hypoxia, as a mechanism that allows continued glycolytic flux (Park et al., 2017). This mechanism may also represent an adaptation strategy in subterranean rodents that allows rapid energy supply in hypoxic environments. Our results also seemed to suggest that Gansu zokor increased the fructose-driven glycolysis in hypoxia. Interestingly, we also observed that Gansu zokors exhibit a simultaneous enhancement of glucose-driven glycolysis and fructose-driven glycolysis in hypoxia II group, while hypoxia I did not significantly change glucose-driven glycolysis. This regulation of glycolysis imply differences in the energy responses of Gansu zokor brain under different hypoxia conditions.

Glucose is the obligatory energy source in the brain, and is transported into the brain *via* GLUT1 (Bélanger et al., 2011; Koch and Weber, 2019; Pragallapati and Manyam, 2019), where it is converted by PFK, PFK is the first irreversible reaction in glucose-driven glycolysis, playing an important role in the control of the glycolysis pathway (Nakajima, 1995; Moreno-Sánchez et al., 2007; Real-Hohn et al., 2010). We found that GLUT1 protein levels were increased in Gansu zokor brain under hypoxia II condition, which is consistent with similar hypoxia responses reported in some tumor cells (Chen et al., 2010; Cretenet et al., 2016). GLUT1 overexpression increases glucose metabolism (Freemerman et al., 2014), indicating that Gansu zokor brain has the capacity for increased glucose-driven glycolysis to cope with hypoxia II. Moreover, the plasma glucose levels of Gansu zokor rose approximately 50% in hypoxia II compared with the levels in normoxia, which consistent with results observed in the naked mole rat, healthy adults, and Oriental river prawn (Newhouse et al., 2017; Sun et al., 2018; Pamenter et al., 2019); however, brain glucose levels in Gansu zokor were significantly decreased under hypoxia II condition. Thus, even with the increase in plasma glucose levels under hypoxia II condition, concentrations of the glucose substrate in the brain decline, suggesting that hypoxia II promotes cerebral glycolysis (Vannucci and Vannucci, 2000; Mergenthaler et al., 2013).

Similar to Gansu zokors, rats showed the same enhancement in glucose-driven glycolysis in response to hypoxia II; however, it is interesting to note that this enhancement in rats was due to the overexpression of both GLUT1 and PFK. We observed that only in rats, *pfk* expression at both the mRNA and its protein levels was significantly increased after exposure to hypoxia II condition; thus, even though its levels in rats was significantly lower than those in Gansu zokor under normoxia, there were no significant differences in *pfk* expression between two species under hypoxia II condition. These results suggest that the enhancement of glucose-driven glycolysis in Gansu zokor brain under hypoxia II condition may be less marked than that in rats. However, rats did not show the same capacity for fructolysis in the brain. This indicates that Gansu zokor has evolved a strategy for coping with hypoxia that is different from that of rats, in that Gansu zokor utilize fructose metabolism to provide energy rapidly. To sum up, Gansu zokor brain may increase both glucose-driven and fructose-driven simultaneously when exposed to hypoxia II, suggesting greater requirement for energy supply in the brain under conditions of hypoxia II of 6.5% O_2_ for 6 h. Finally, it is important to note that the elevation of plasma glucose induced in Gansu zokor exposed to hypoxia is considerably greater than the increase in plasma fructose, which is consistent with the pattern reported in the naked mole rat (Park et al., 2017). Furthermore, whether in normoxia or hypoxia, the levels of glucose in Gansu zokor are much higher than the fructose levels, indicating that, on the basis of glucose as the main metabolic substrate, fructose is used to accelerate the supply of energy in Gansu zokor.

### Study Limitations

This study has some limitations. First, we did not collect the oxygen concentration data for Gansu zokor in their wild underground burrows at different depths, seasons, and weather; our selection of the hypoxia treatment conditions (hypoxia I: 44 h at 10.5% O_2_; hypoxia II: 6 h at 6.5% O_2_) was based mainly on laboratory tests of hypoxia tolerance in Gansu zokor and previous studies of *Spalax* (Shams et al., 2005; Band et al., 2010; Moskovitz et al., 2012; Yan et al., 2012). Second, the duration time of hypoxic II treatment can ensure the survival of rats for tissue sampling, and the hypoxic I treatment is a relatively long-mild form of hypoxia; these two hypoxic treatment conditions, including oxygen concentration and duration time, reflect the different hypoxic environments that may occur in the burrows inhabited by Gansu zokor in the wild; but even these rigorous simulations can not totally represent the burrow environment. Besides, more hypoxic experimental groups will be required to perfect the design of experiment. Finally, we have investigated glycolysis differences in Gansu zokor and Sprague-Dawley rat; our aim was to reveal the hypoxic tolerance mechanism in subterranean rodent through exploring the different hypoxic response in hypoxia-tolerant subterranean Gansu zokor and hypoxia-intolerant surface rat. However, in the current study, although Sprague-Dawley rats are non-burrowing rodents, they are not the best controls because of not living in the wild environment; therefore, our discussion of the comparison between two species remains limited.

## Conclusion

Gansu zokor has evolved the capacity to utilize fructose to fuel the brain on the basis of glucose metabolism. This metabolic rewiring of glycolysis seems to be an adaptive that underlies the hypoxia tolerance of this species. Gansu zokor exhibited significantly higher levels of GLUT5 and KHK proteins, as well as increased KHK enzymatic activity compared with the levels in the hypoxia-intolerant surface-dwelling rat. GLUT5 and KHK represent molecular signatures of fructose metabolism that are also upregulated by hypoxia stress. Thus, these results reveal the mechanism underlying hypoxia tolerance in Gansu zokor. Further studies of energy metabolism in “natural” hypoxia tolerance in an animal model will provide an important supplement to hypoxia adaptation studies and highlight potential strategies to mitigate hypoxic brain damage, which can provide theoretical basis and novel strategies for hypoxic brain injury treatment of human beings.

## Data Availability Statement

The raw data supporting the conclusions of this article will be made available by the authors, without undue reservation.

## Ethics Statement

The animal study was reviewed and approved by Animal Management Committee and Ethical Review Committee of Experimental Animal Welfare, Shaanxi Normal University.

## Author Contributions

JGL and JH designed the study. JYL wrote the manuscript with help of JGL. LF, LC, JK, and XW contributed to experimental work. JYL, YH, JG, and ZH performed the data analysis. All authors contributed to the revision of the manuscript and approved the final version of the manuscript.

## Conflict of Interest

The authors declare that the research was conducted in the absence of any commercial or financial relationships that could be construed as a potential conflict of interest.
