# Supplementary figures and images for: The mTORC1/eIF4E/HIF-1α Pathway Mediates Glycolysis to Support Brain Hypoxia Resistance in the Gansu Zokor, Eospalax cansus
Source: Front Physiol. 2021 Feb 23;12:626240. doi: 10.3389/fphys.2021.626240 (PMC7940537; doi:10.3389/fphys.2021.626240)

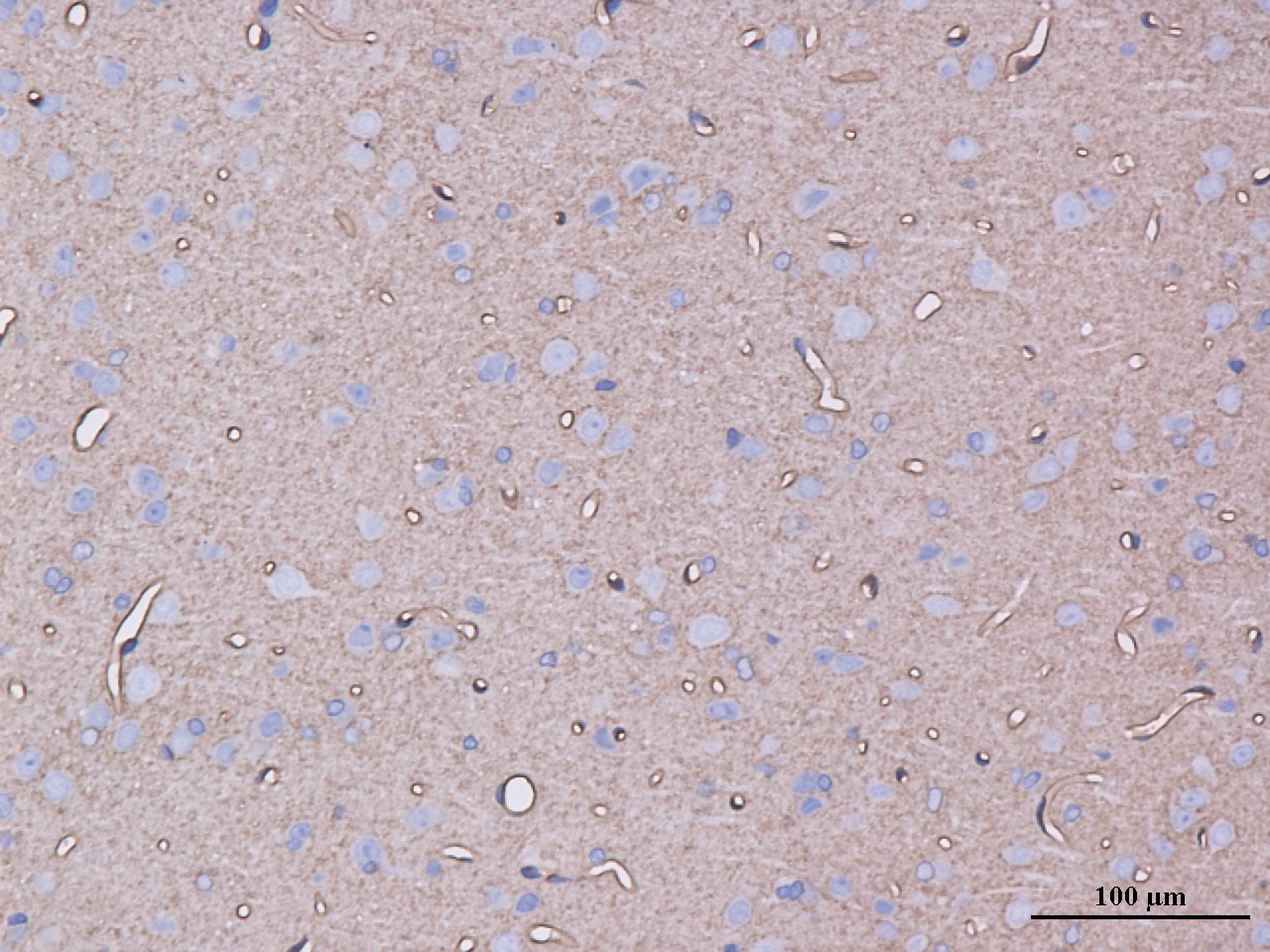

Supplement: Supplementary file 1 [file Data_Sheet_1.ZIP › Immunohistochemistry-GLUT1/1.Figure 5E. SD rat-GLUT1-21%.tif]

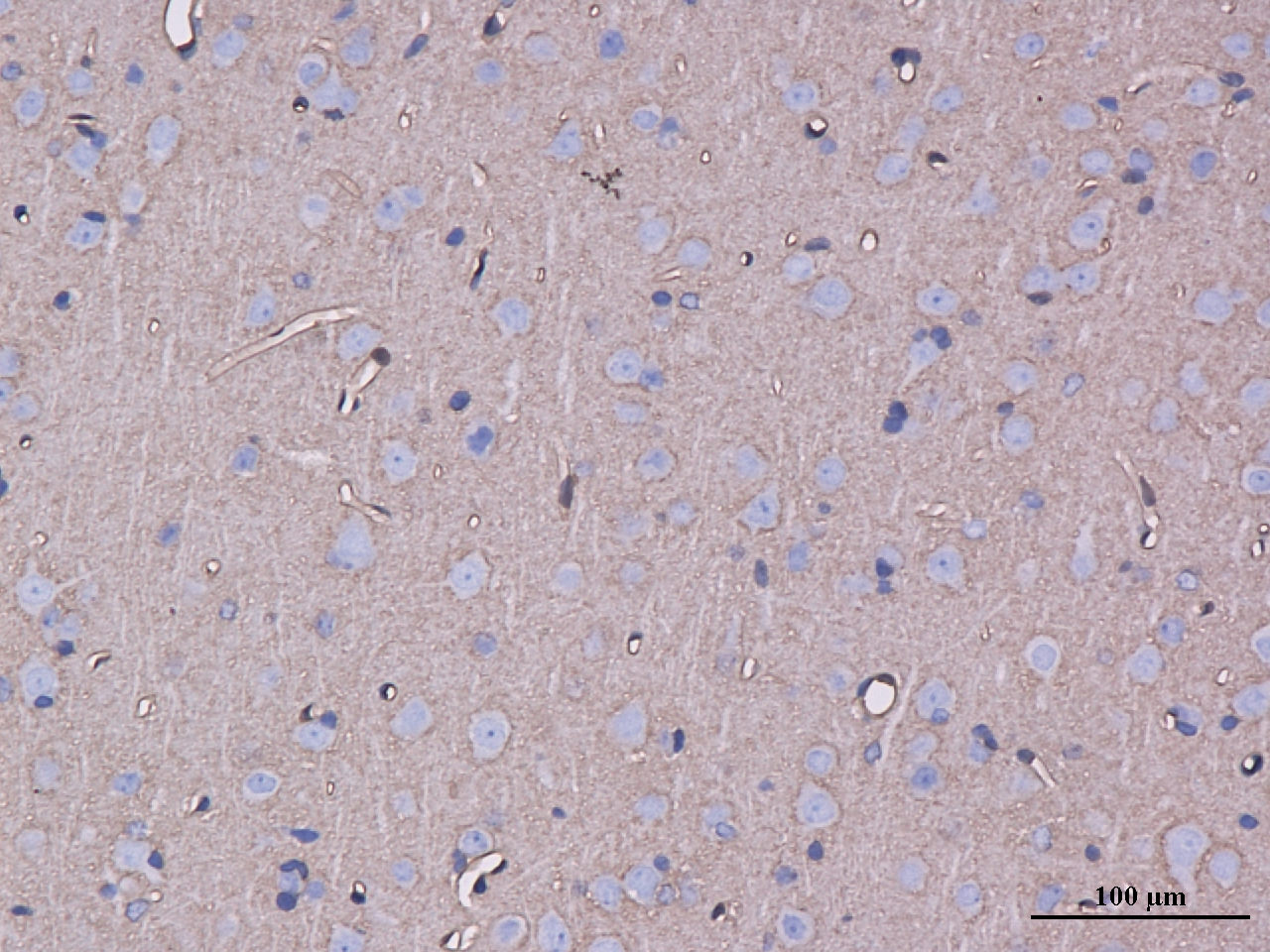

Supplement: Supplementary file 1 [file Data_Sheet_1.ZIP › Immunohistochemistry-GLUT1/2.Figure 5E. SD rat-GLUT1-10.5%.tif]

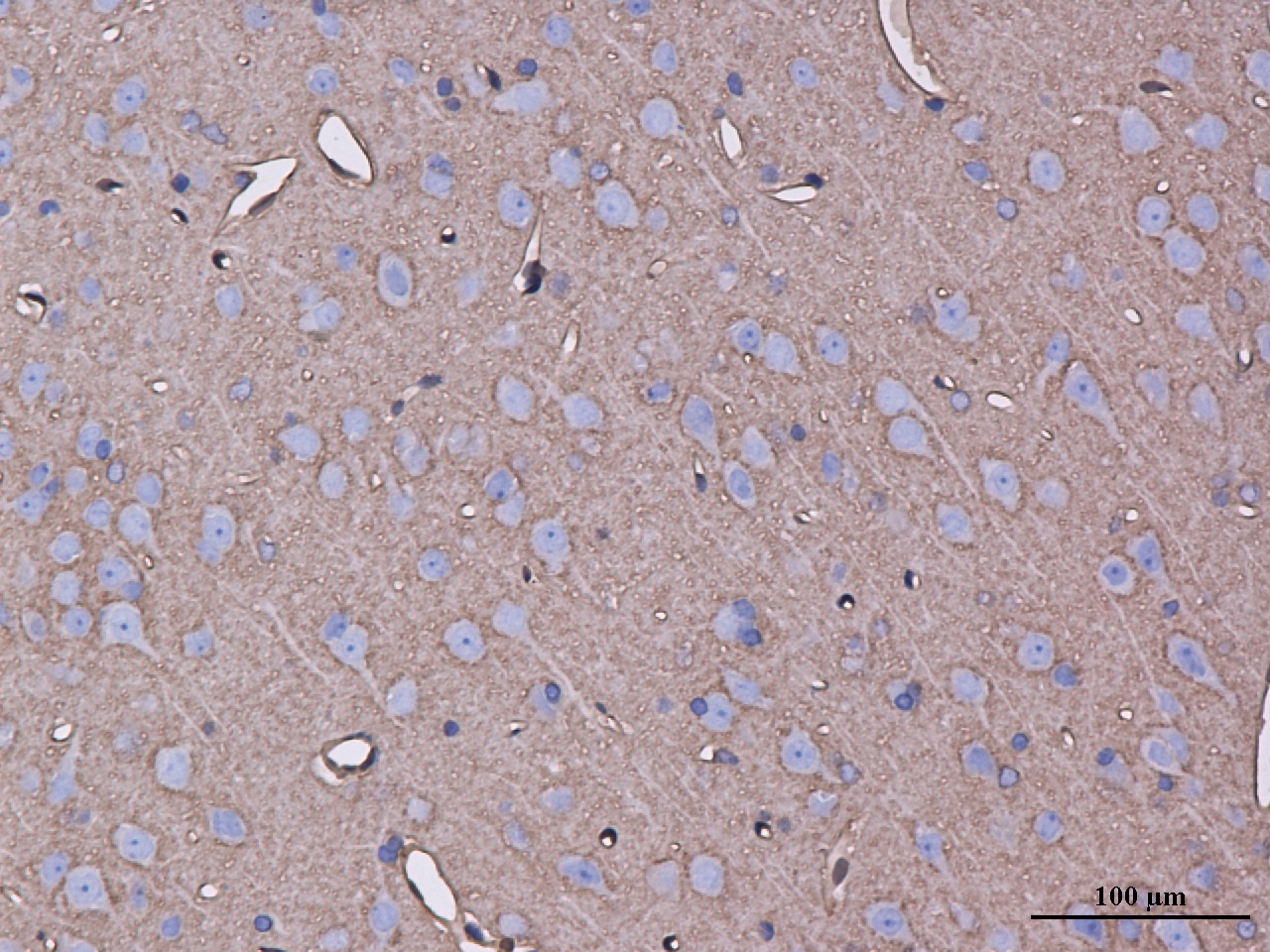

Supplement: Supplementary file 1 [file Data_Sheet_1.ZIP › Immunohistochemistry-GLUT1/3.Figure 5E. SD rat-GLUT1-6.5%.tif]

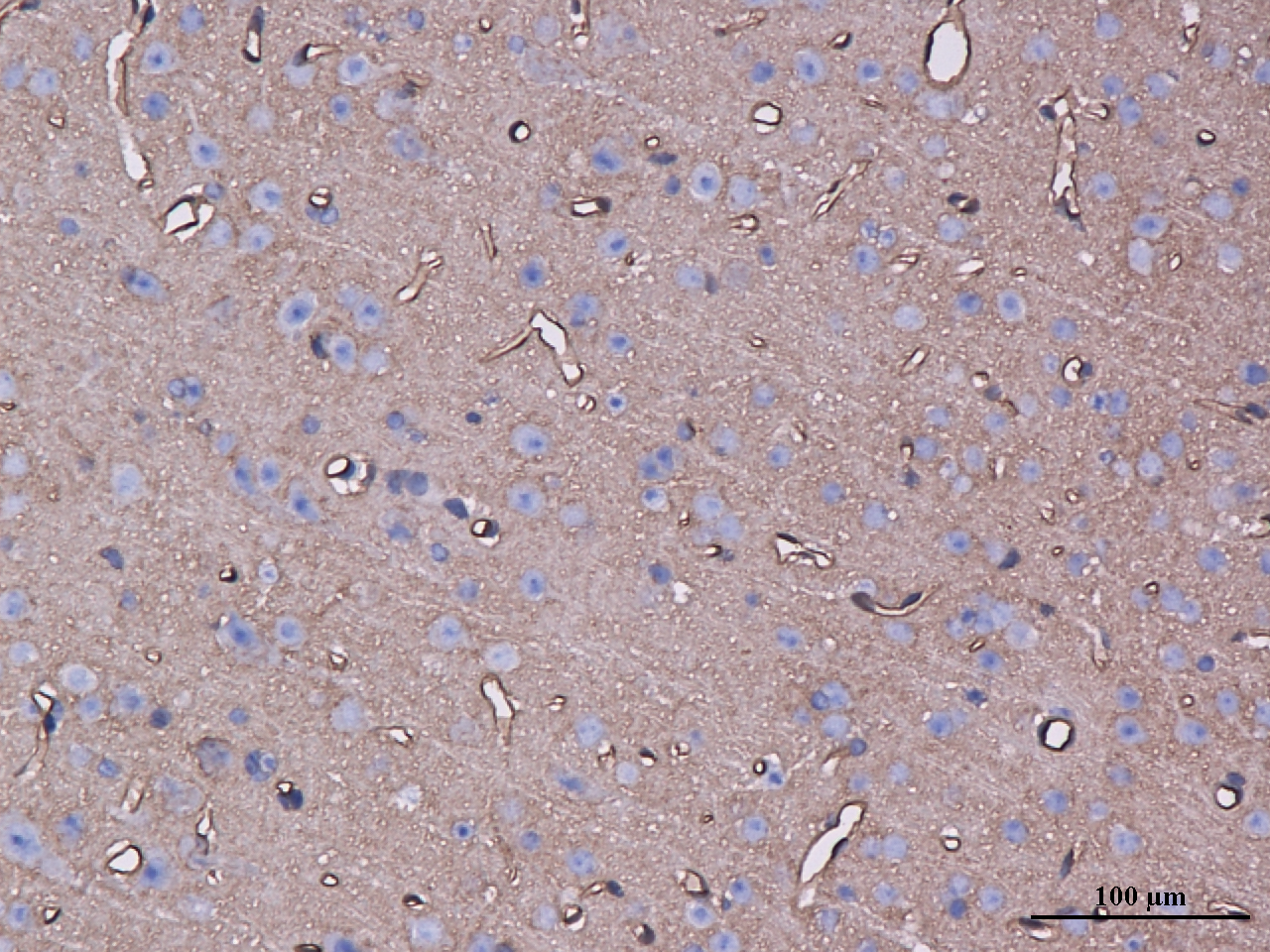

Supplement: Supplementary file 1 [file Data_Sheet_1.ZIP › Immunohistochemistry-GLUT1/4.Figure 5E. zokor-GLUT1-21%.tif]

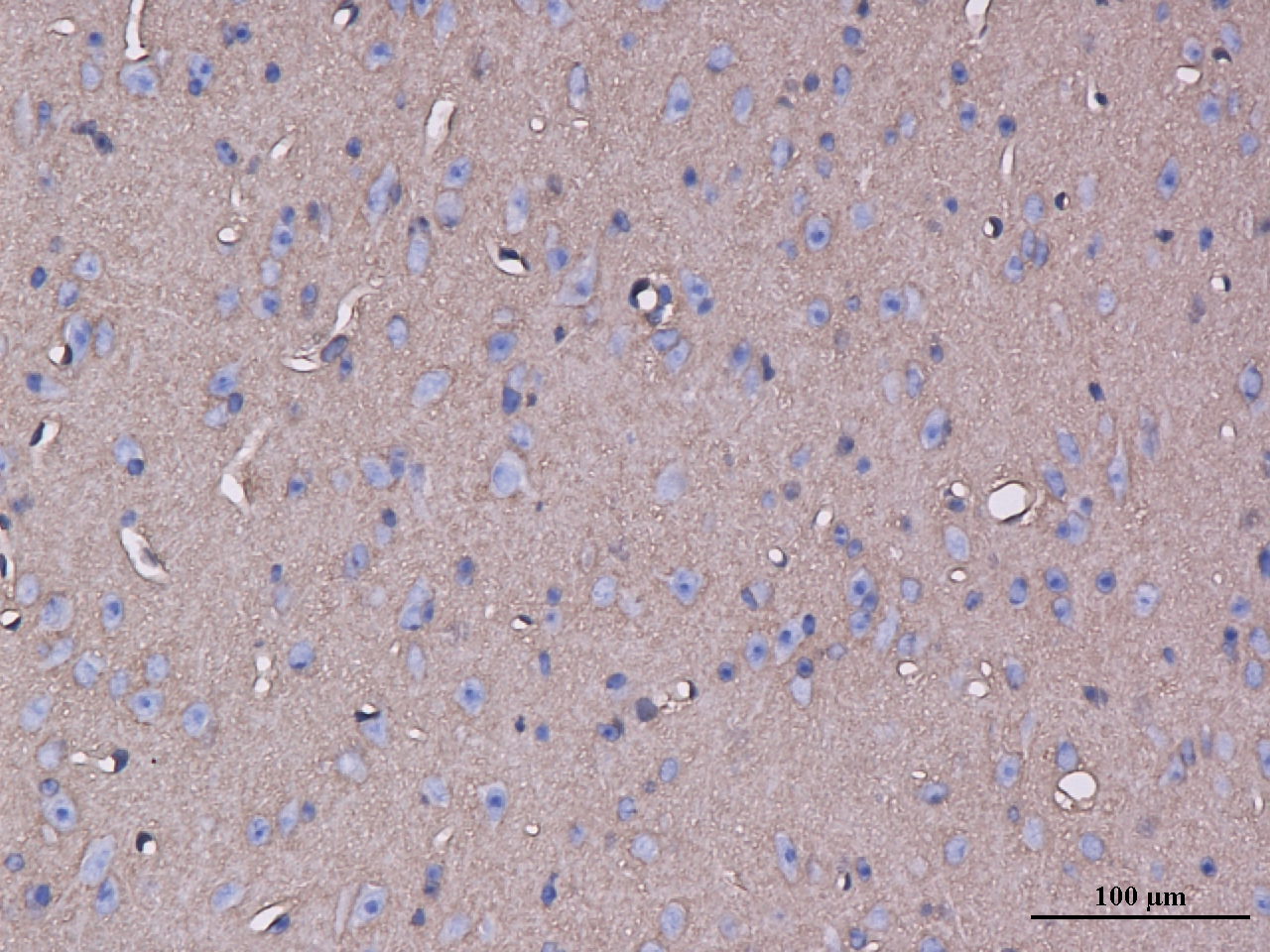

Supplement: Supplementary file 1 [file Data_Sheet_1.ZIP › Immunohistochemistry-GLUT1/5.Figure 5E. zokor-GLUT1-10.5%.tif]

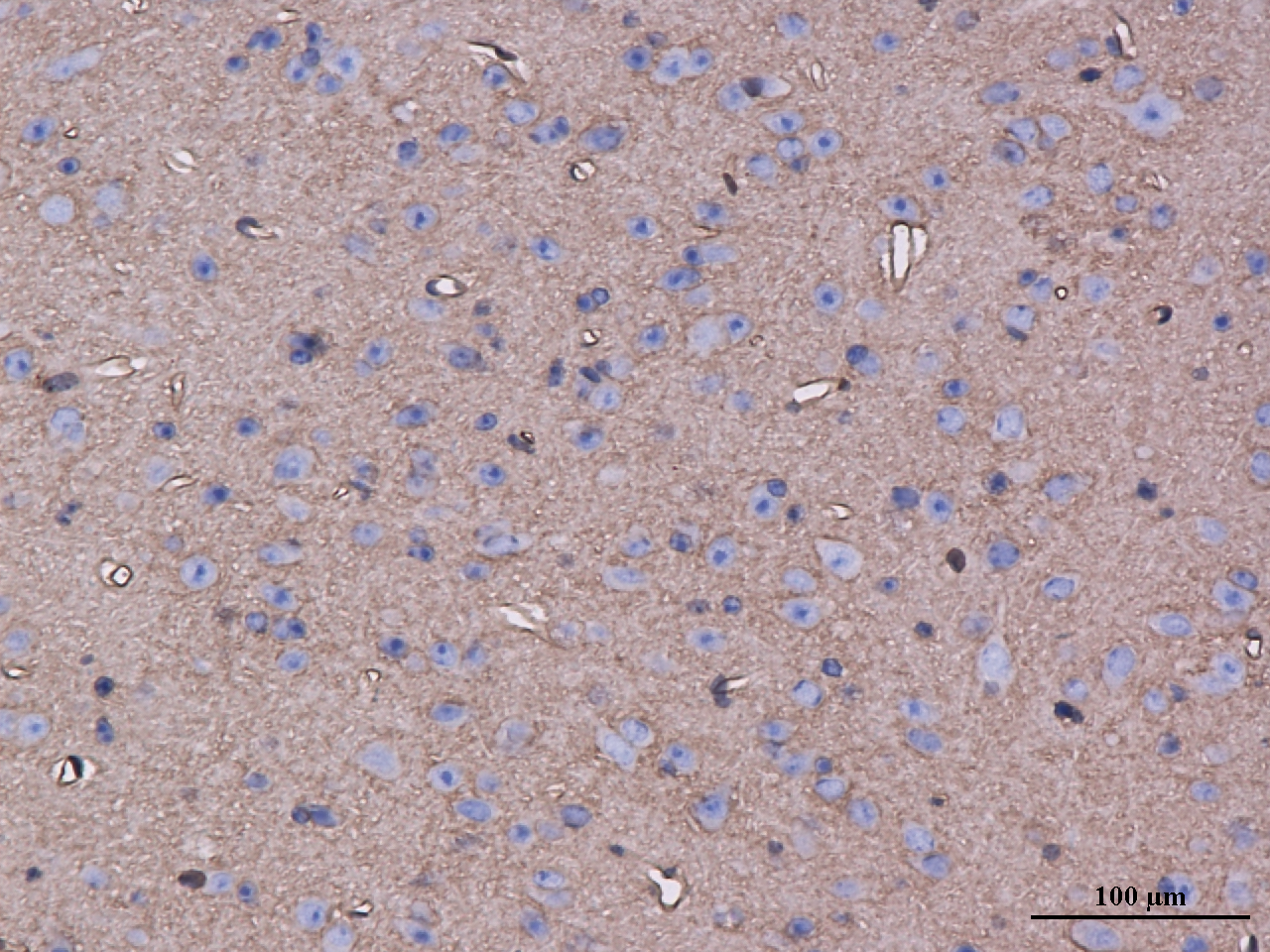

Supplement: Supplementary file 1 [file Data_Sheet_1.ZIP › Immunohistochemistry-GLUT1/6.Figure 5E. zokor-GLUT1-6.5%.tif]

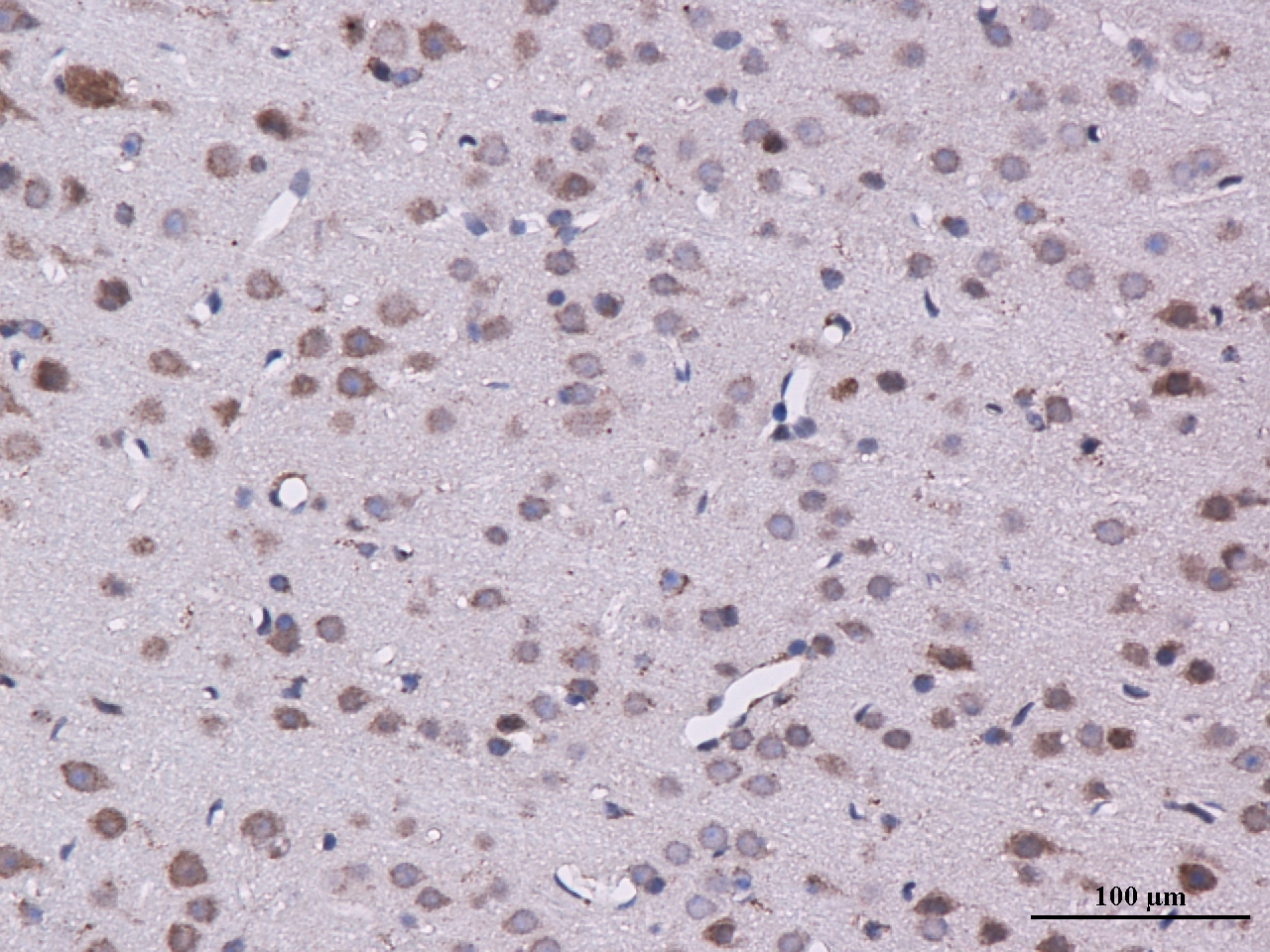

Supplement: Supplementary file 2 [file Data_Sheet_2.ZIP › Immunohistochemistry-PFK/10.Figure 5E. zokor-PFK-21%.tif]

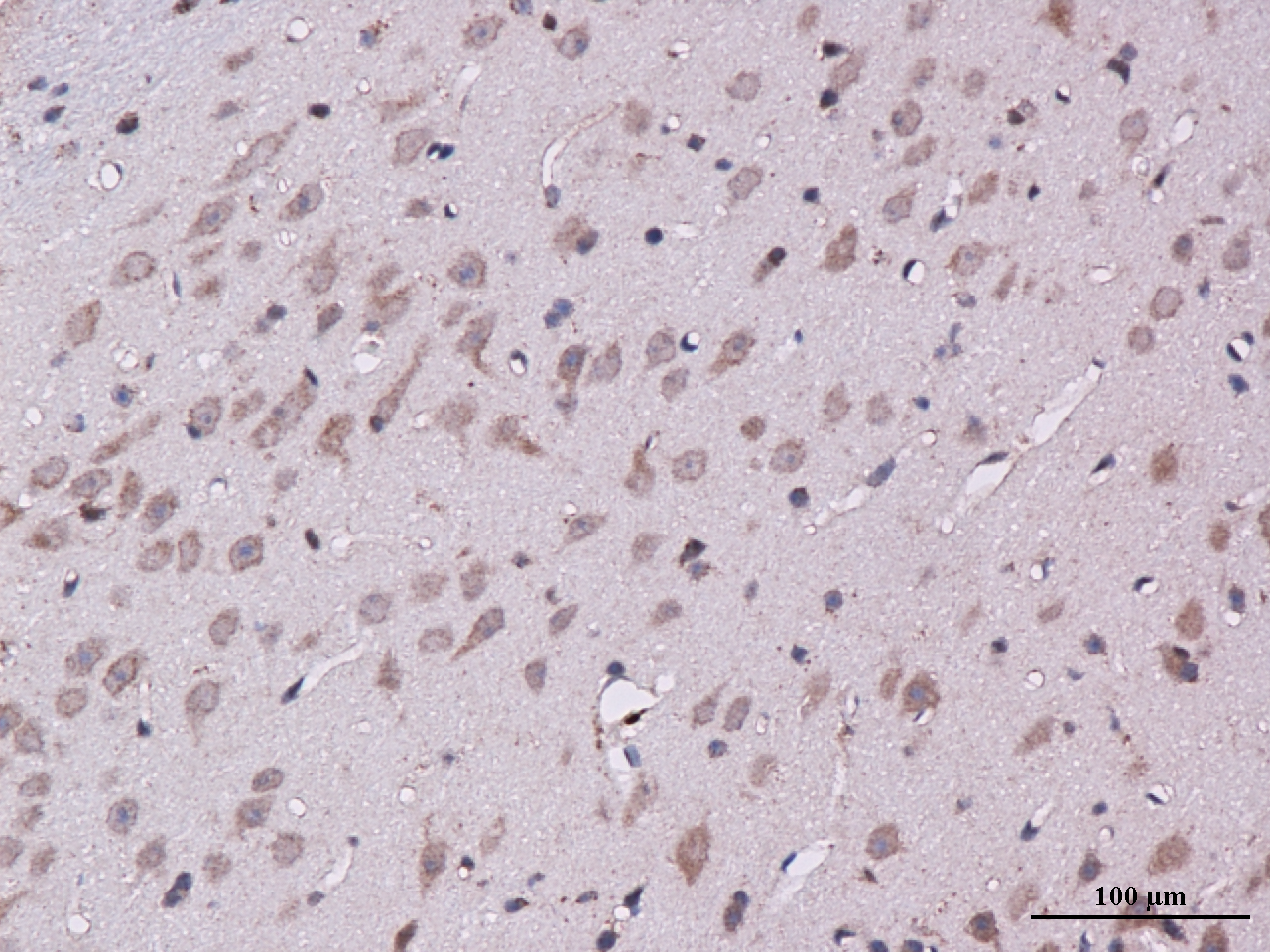

Supplement: Supplementary file 2 [file Data_Sheet_2.ZIP › Immunohistochemistry-PFK/11.Figure 5E. zokor-PFK-10.5%.tif]

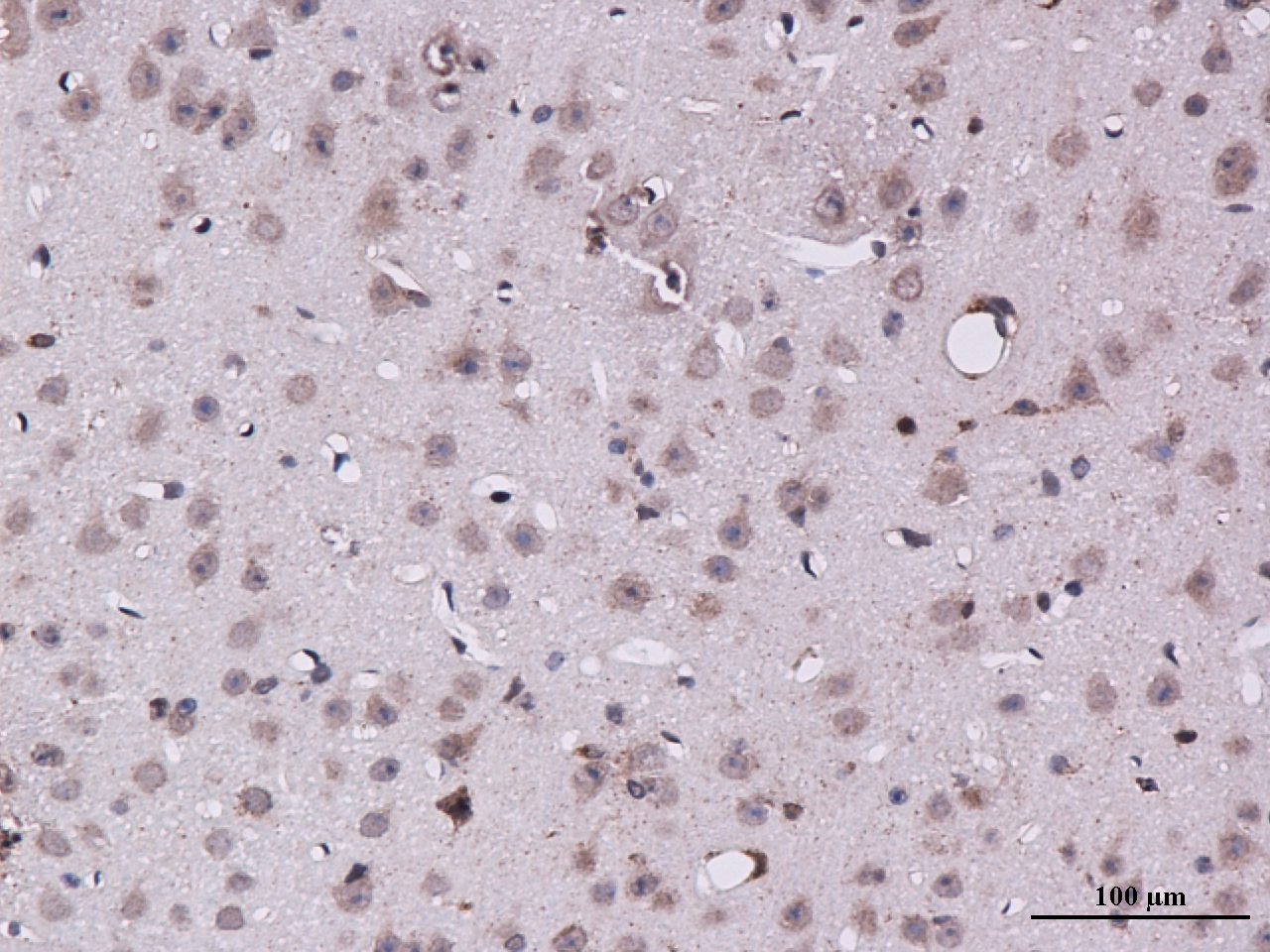

Supplement: Supplementary file 2 [file Data_Sheet_2.ZIP › Immunohistochemistry-PFK/12.Figure 5E. zokor-PFK-6.5%.tif]

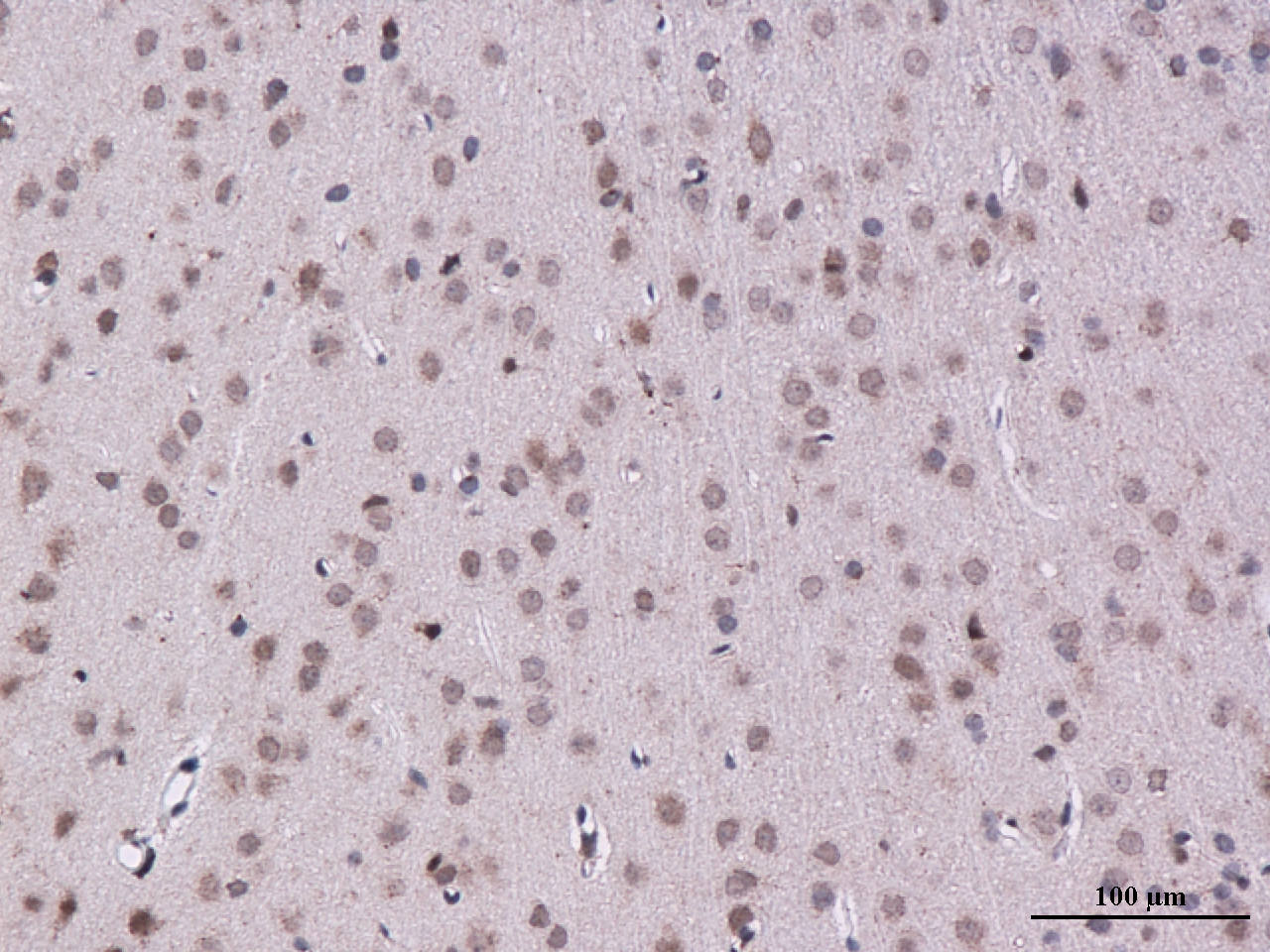

Supplement: Supplementary file 2 [file Data_Sheet_2.ZIP › Immunohistochemistry-PFK/7.Figure 5E. SD rat-PFK-21%.tif]

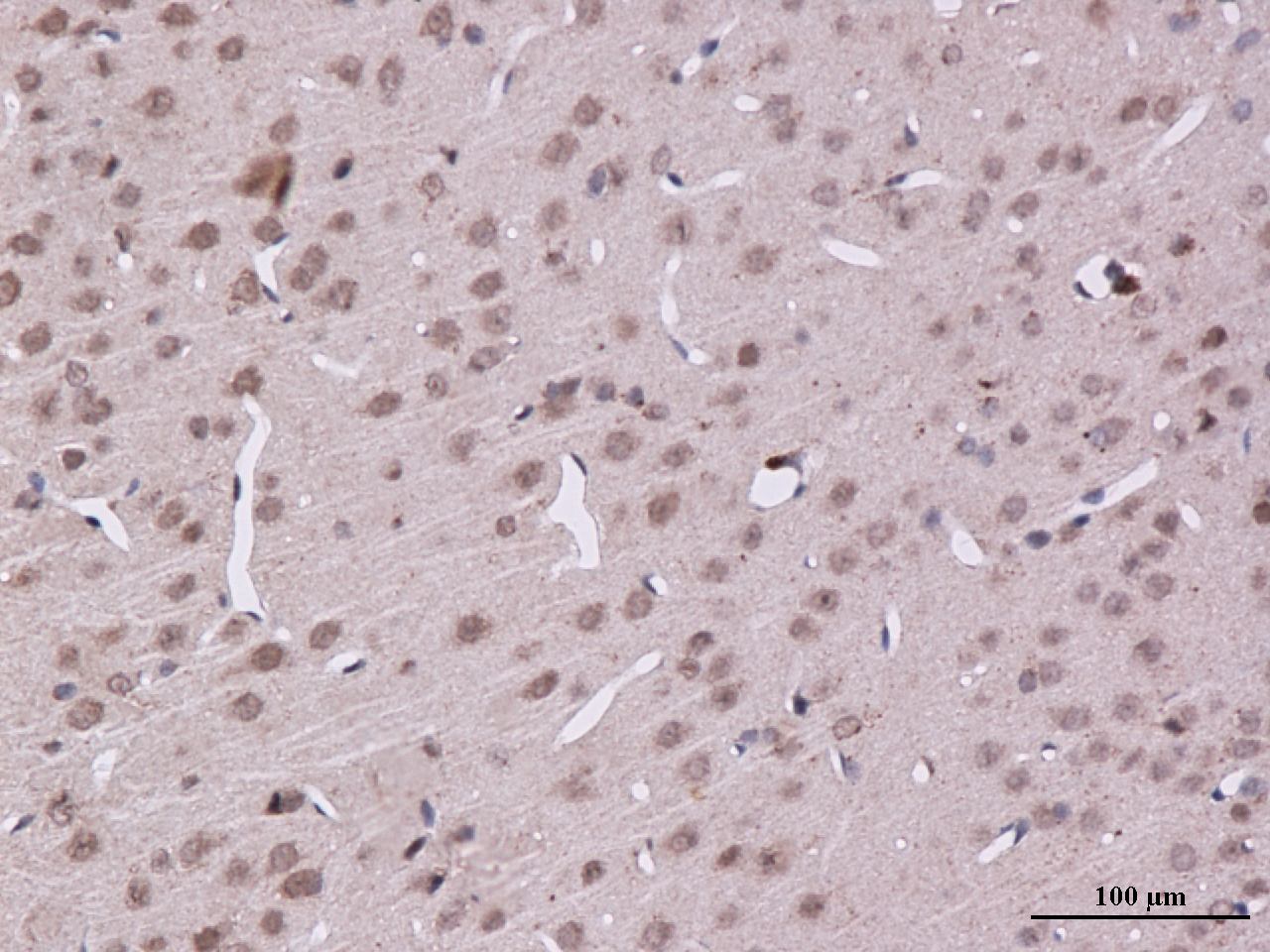

Supplement: Supplementary file 2 [file Data_Sheet_2.ZIP › Immunohistochemistry-PFK/8.Figure 5E. SD rat-PFK-10.5%.tif]

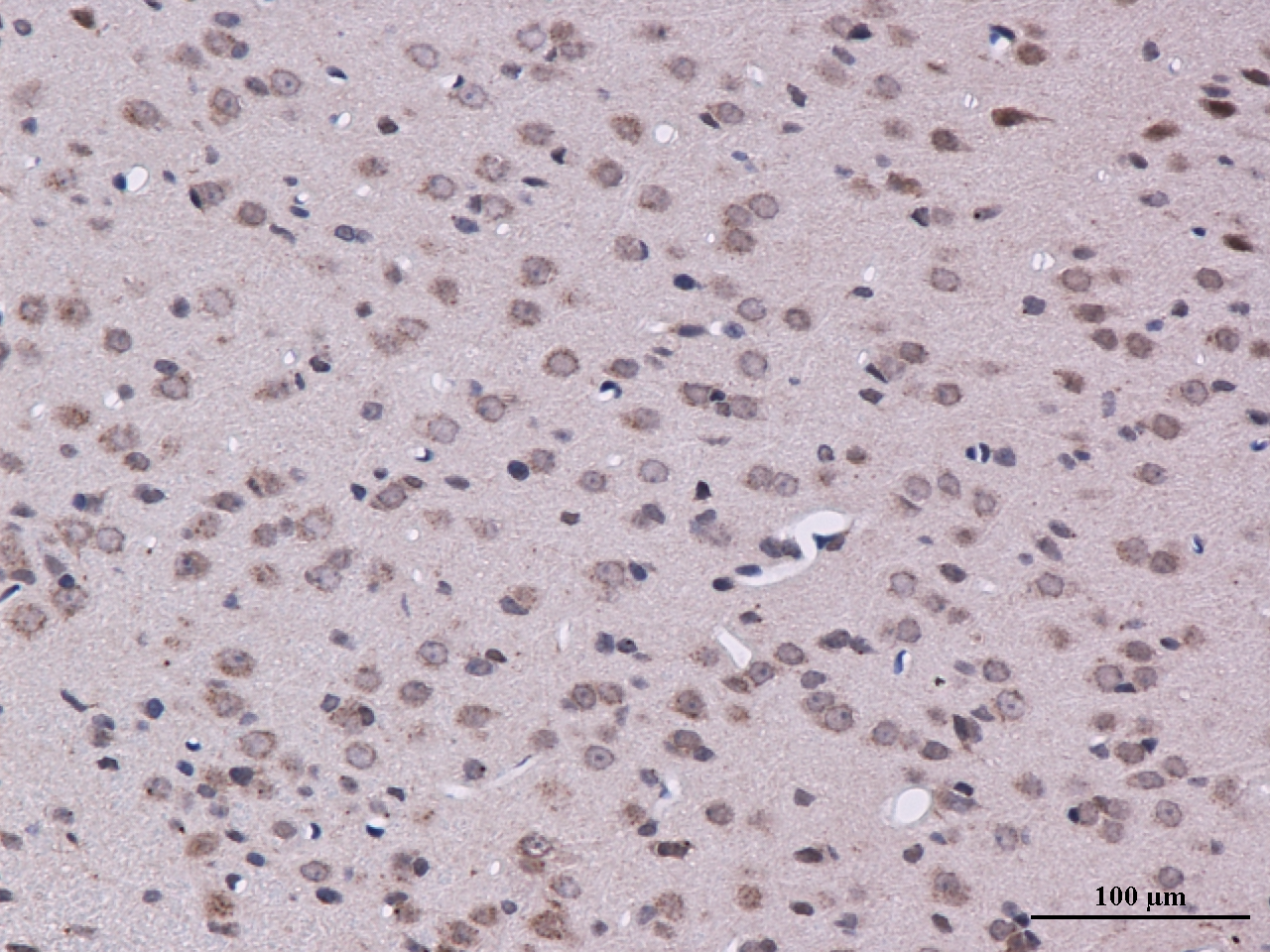

Supplement: Supplementary file 2 [file Data_Sheet_2.ZIP › Immunohistochemistry-PFK/9.Figure 5E. SD rat-PFK-6.5%.tif]

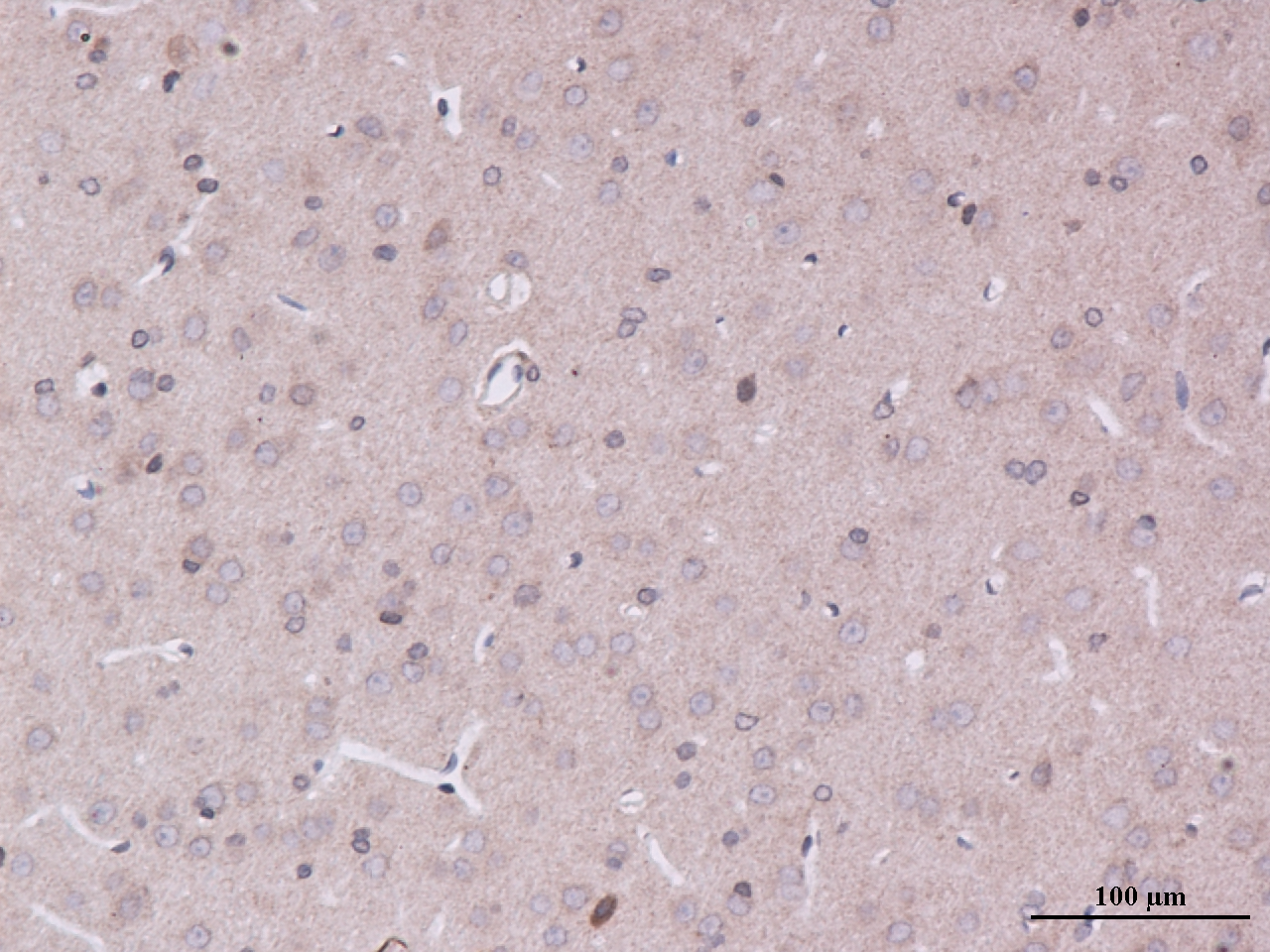

Supplement: Supplementary file 3 [file Data_Sheet_3.ZIP › Immunohistochemistry-GLUT5/13.Figure 5E. SD rat-GLUT5-21%.tif]

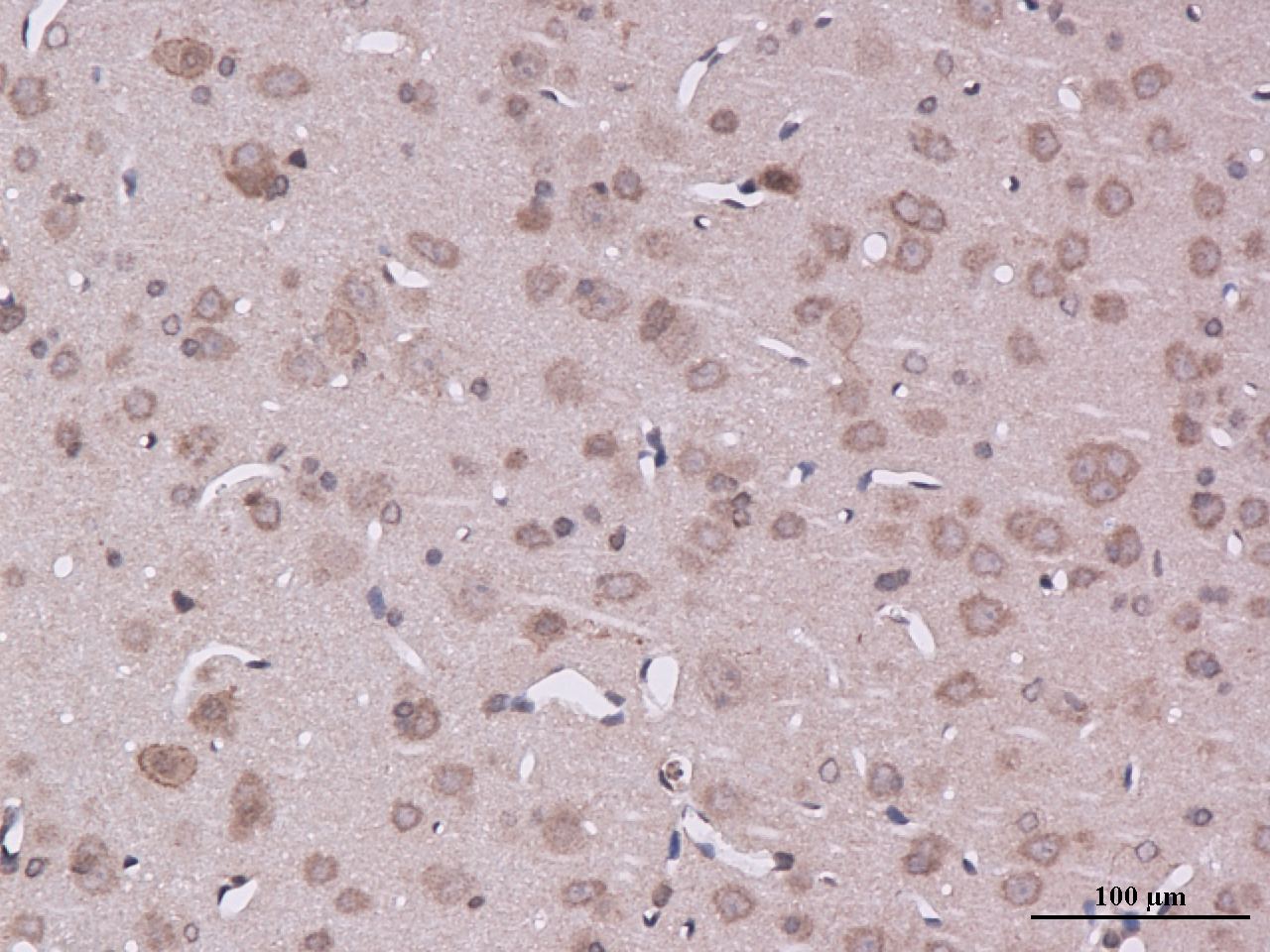

Supplement: Supplementary file 3 [file Data_Sheet_3.ZIP › Immunohistochemistry-GLUT5/14.Figure 5E. SD rat-GLUT5-10.5%.tif]

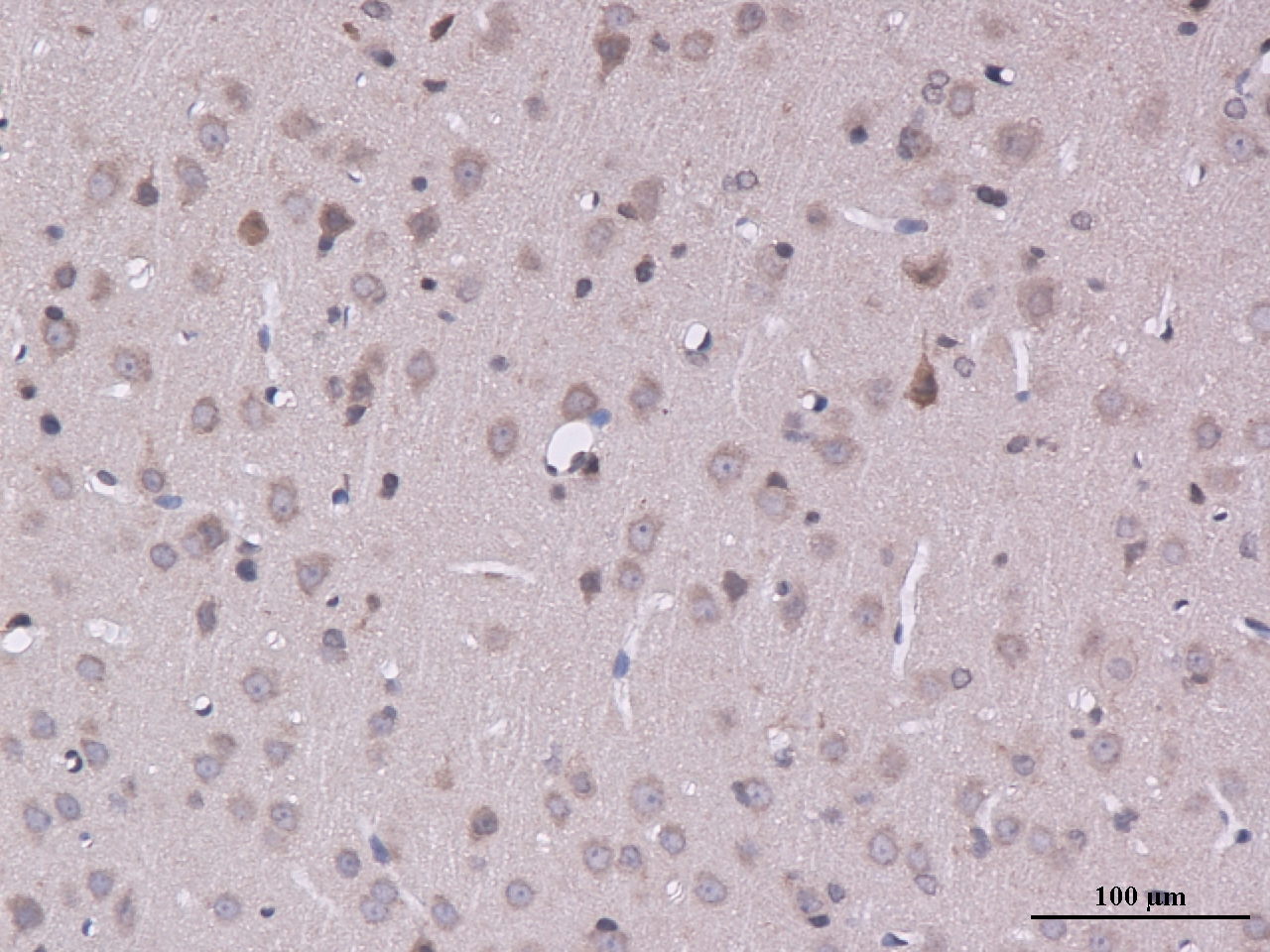

Supplement: Supplementary file 3 [file Data_Sheet_3.ZIP › Immunohistochemistry-GLUT5/15.Figure 5E. SD rat-GLUT5-6.5%.tif]

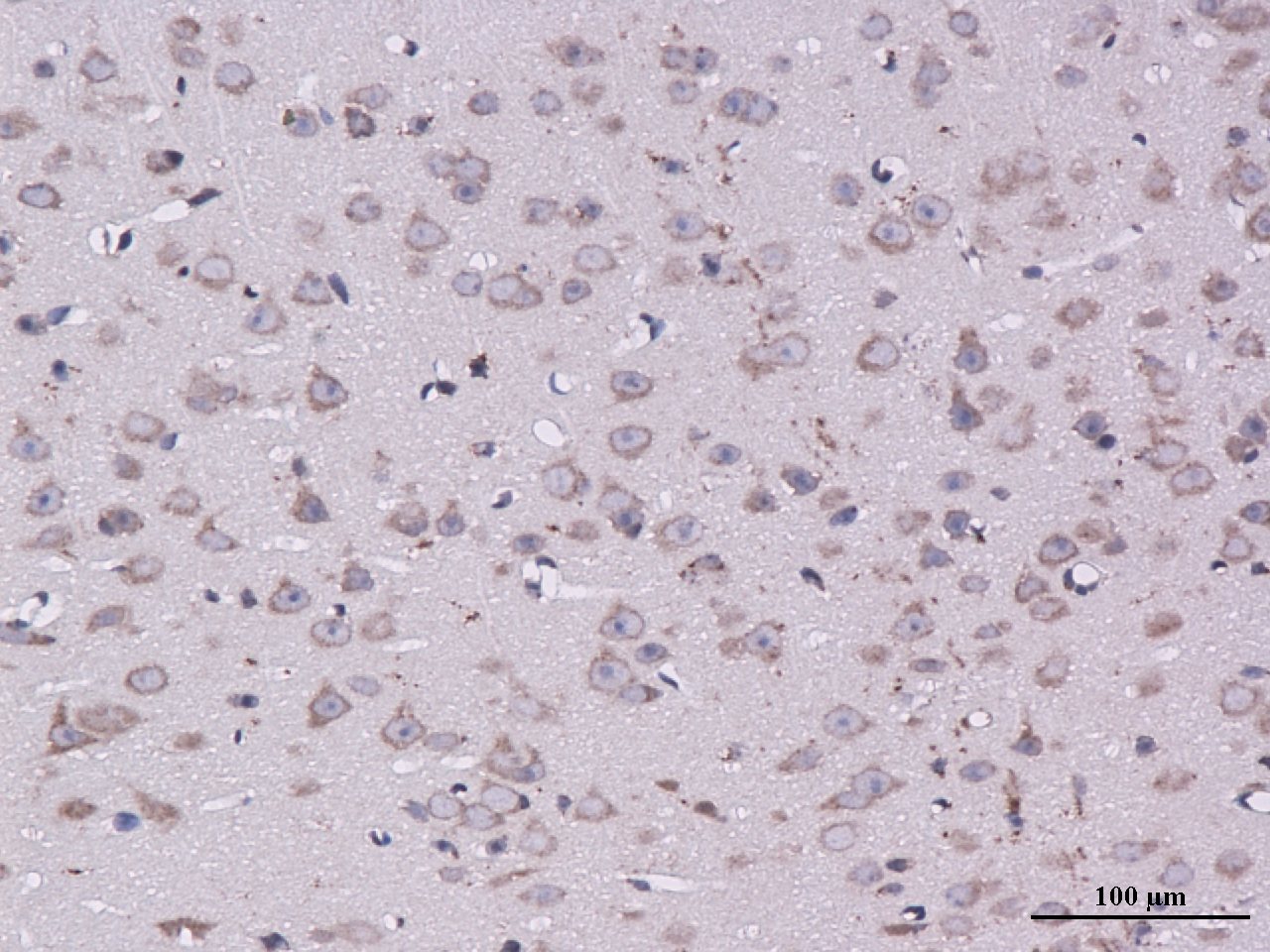

Supplement: Supplementary file 3 [file Data_Sheet_3.ZIP › Immunohistochemistry-GLUT5/16.Figure 5E. zokor-GLUT5-21%.tif]

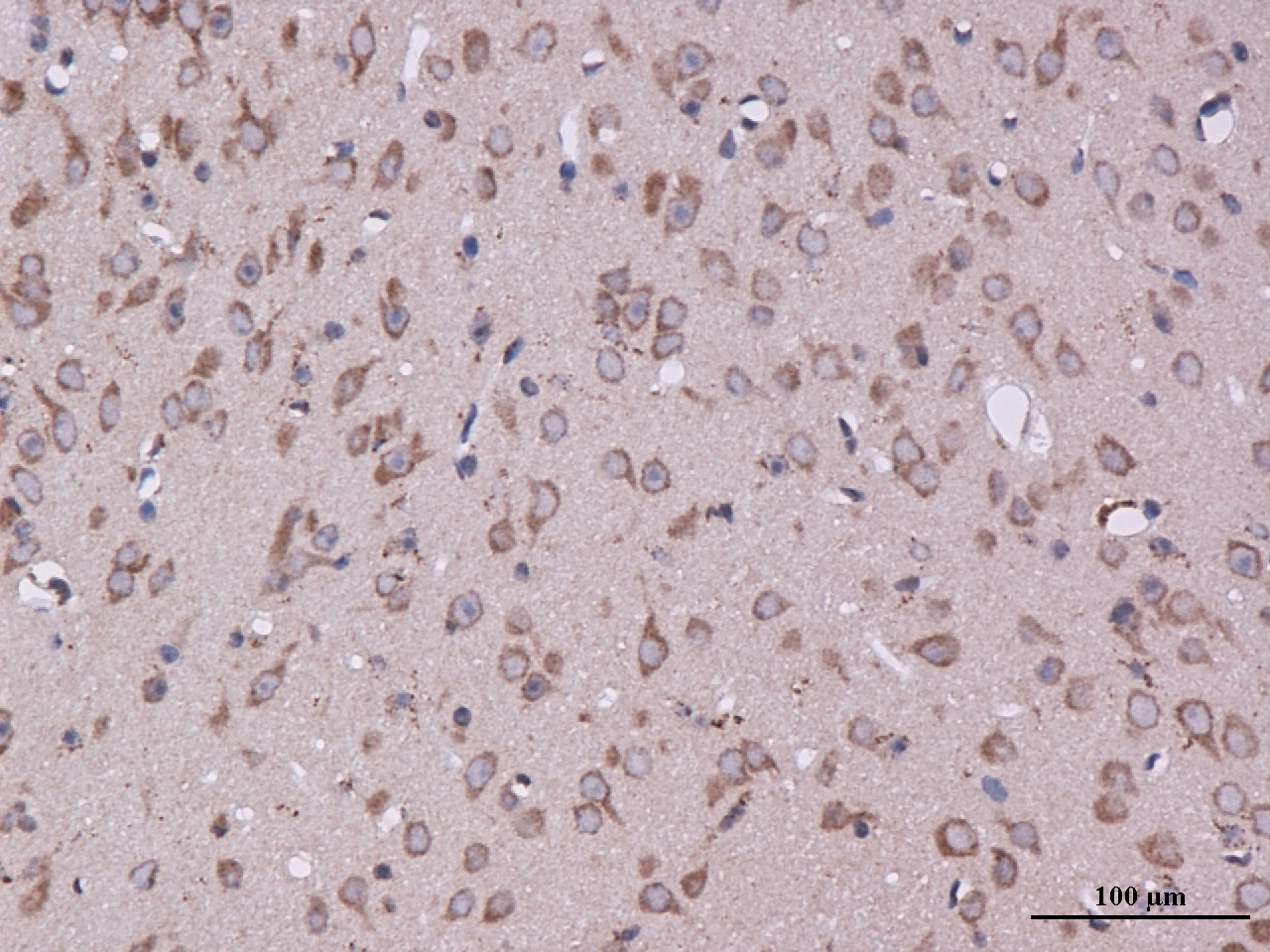

Supplement: Supplementary file 3 [file Data_Sheet_3.ZIP › Immunohistochemistry-GLUT5/17.Figure 5E. zokor-GLUT5-10.5%.tif]

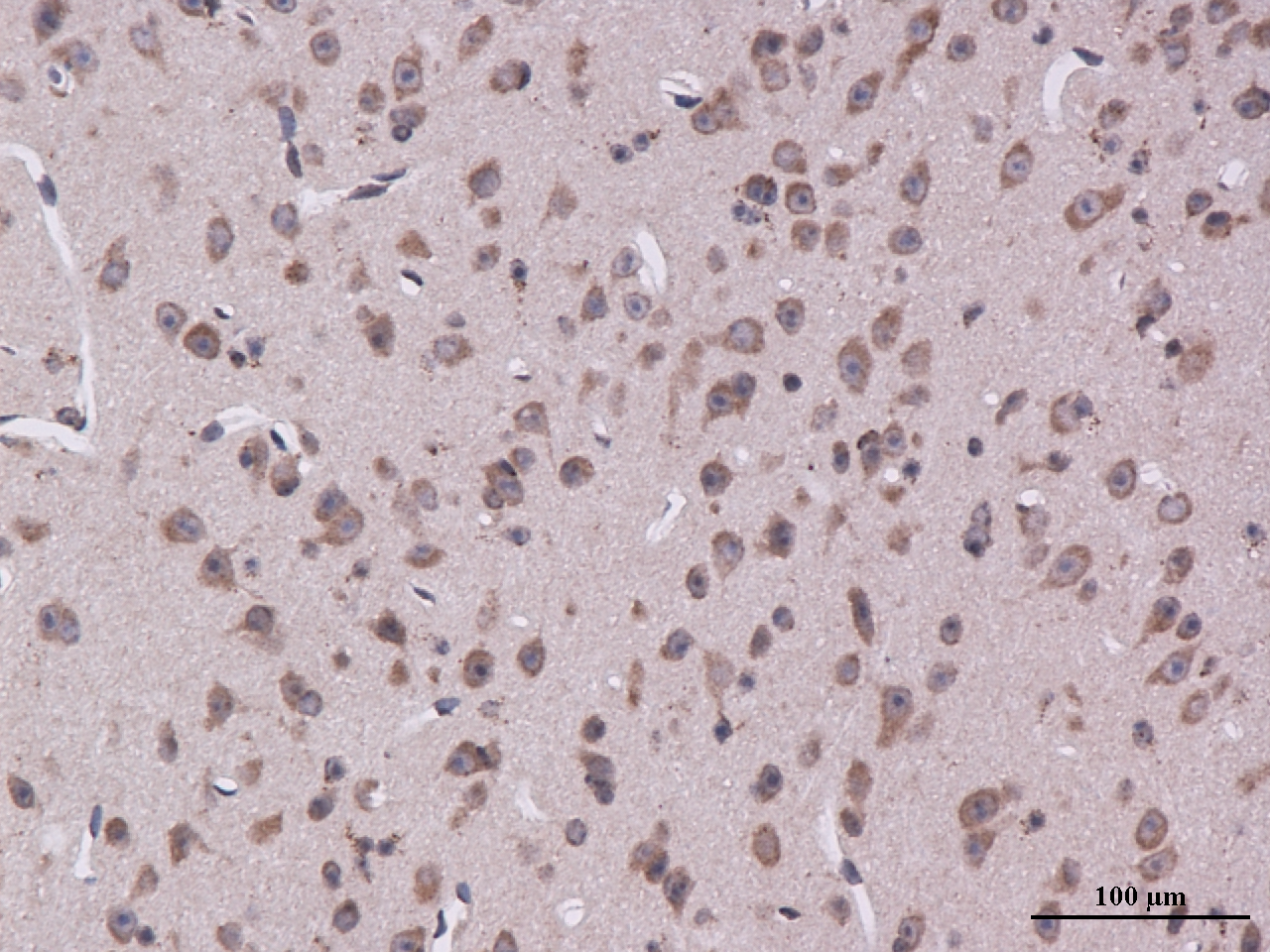

Supplement: Supplementary file 3 [file Data_Sheet_3.ZIP › Immunohistochemistry-GLUT5/18.Figure 5E. zokor-GLUT5-6.5%.tif]

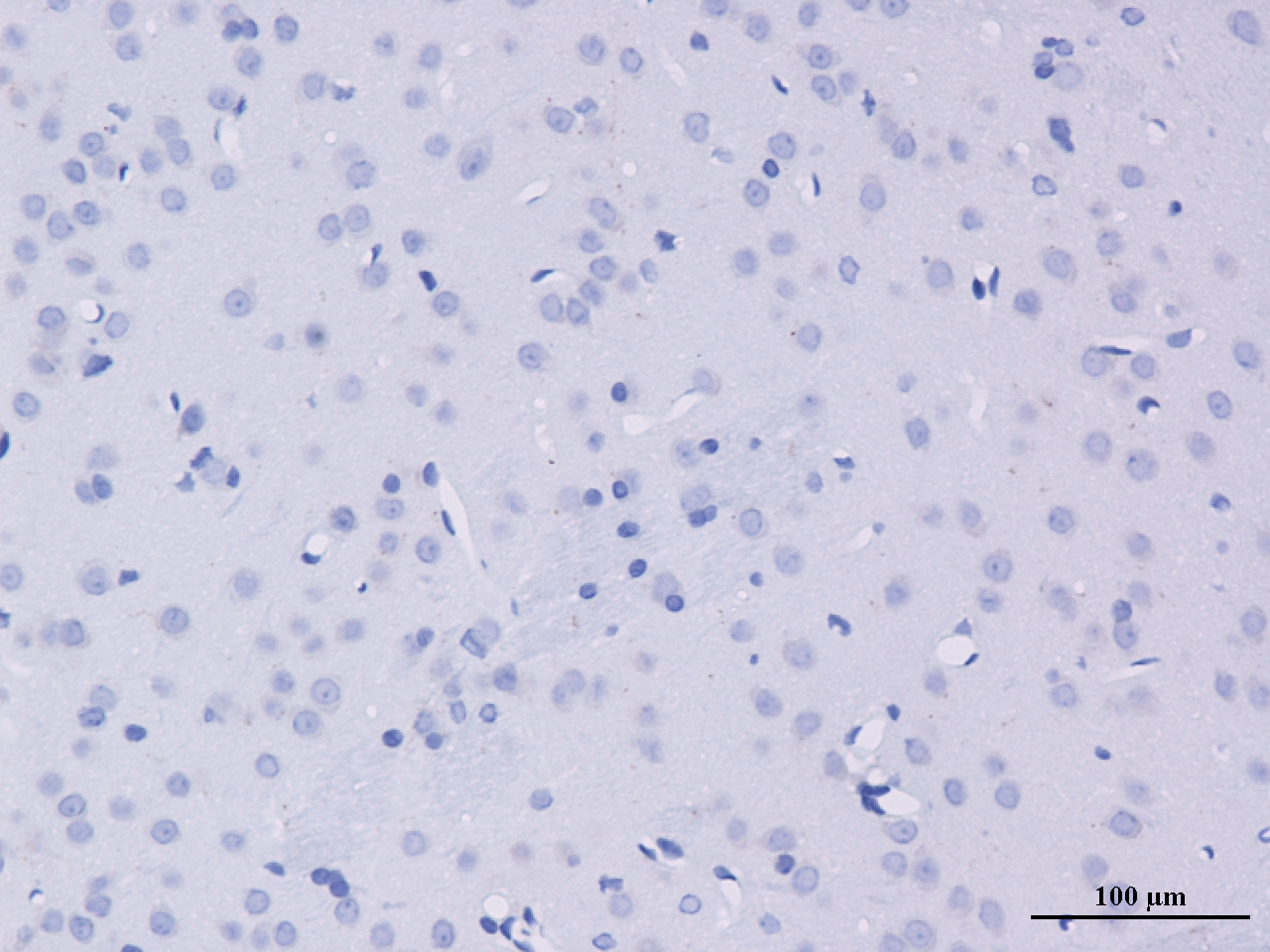

Supplement: Supplementary file 4 [file Data_Sheet_4.ZIP › Immunohistochemistry-KHK/19.Figure 5E. SD rat-KHK-21%.tif]

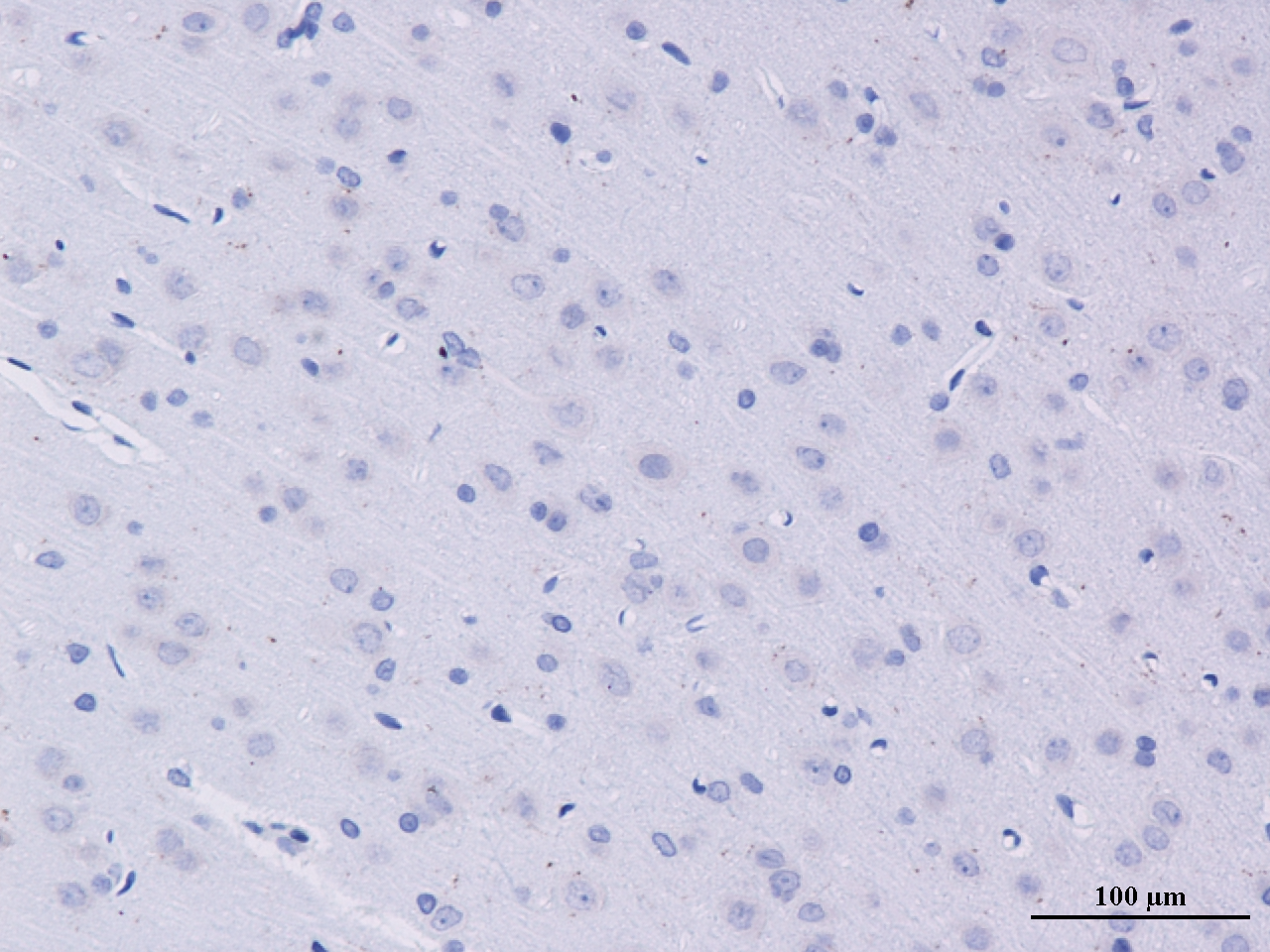

Supplement: Supplementary file 4 [file Data_Sheet_4.ZIP › Immunohistochemistry-KHK/20.Figure 5E. SD rat-KHK-10.5%.tif]

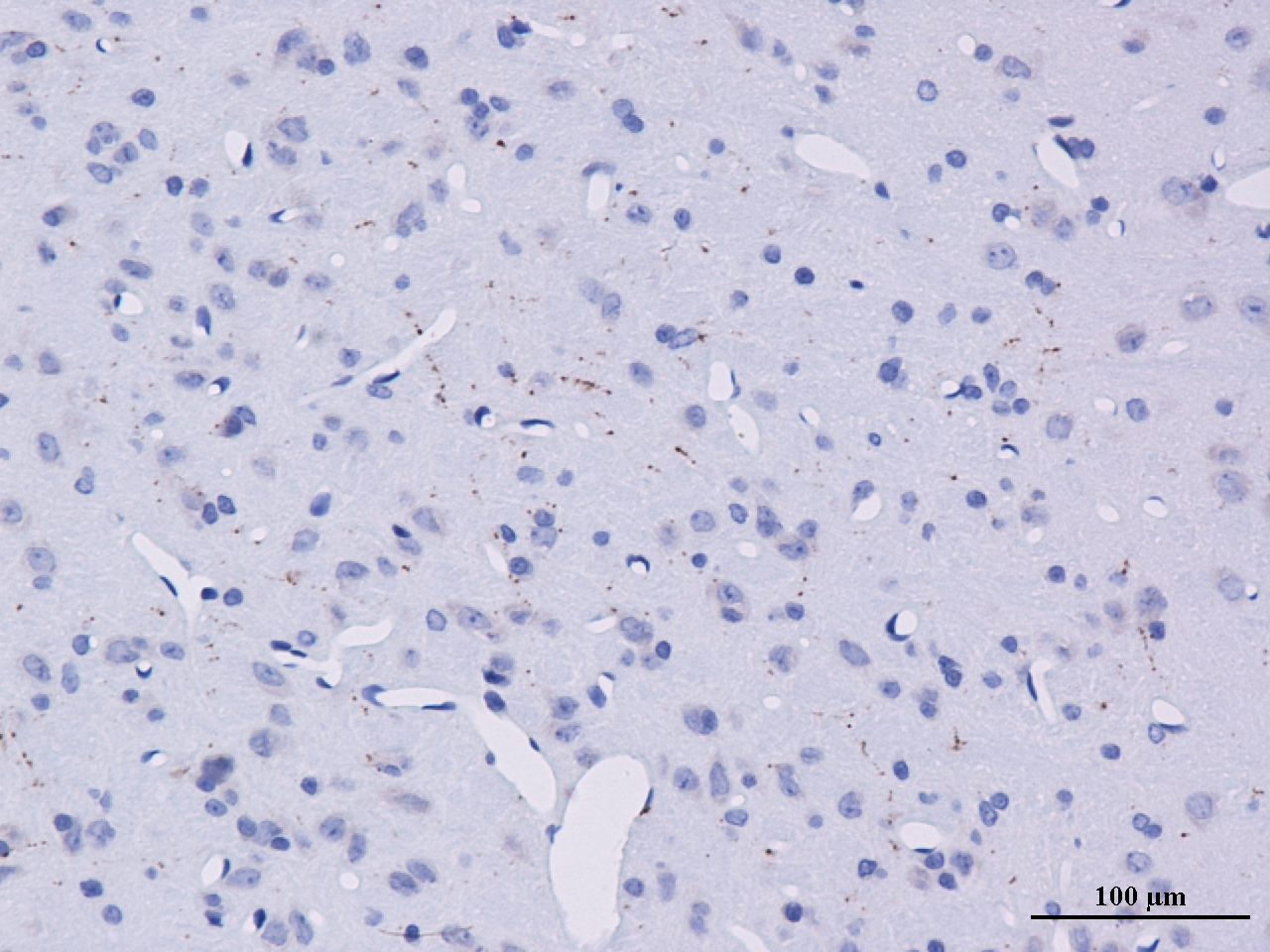

Supplement: Supplementary file 4 [file Data_Sheet_4.ZIP › Immunohistochemistry-KHK/21.Figure 5E. SD rat-KHK-6.5%.tif]

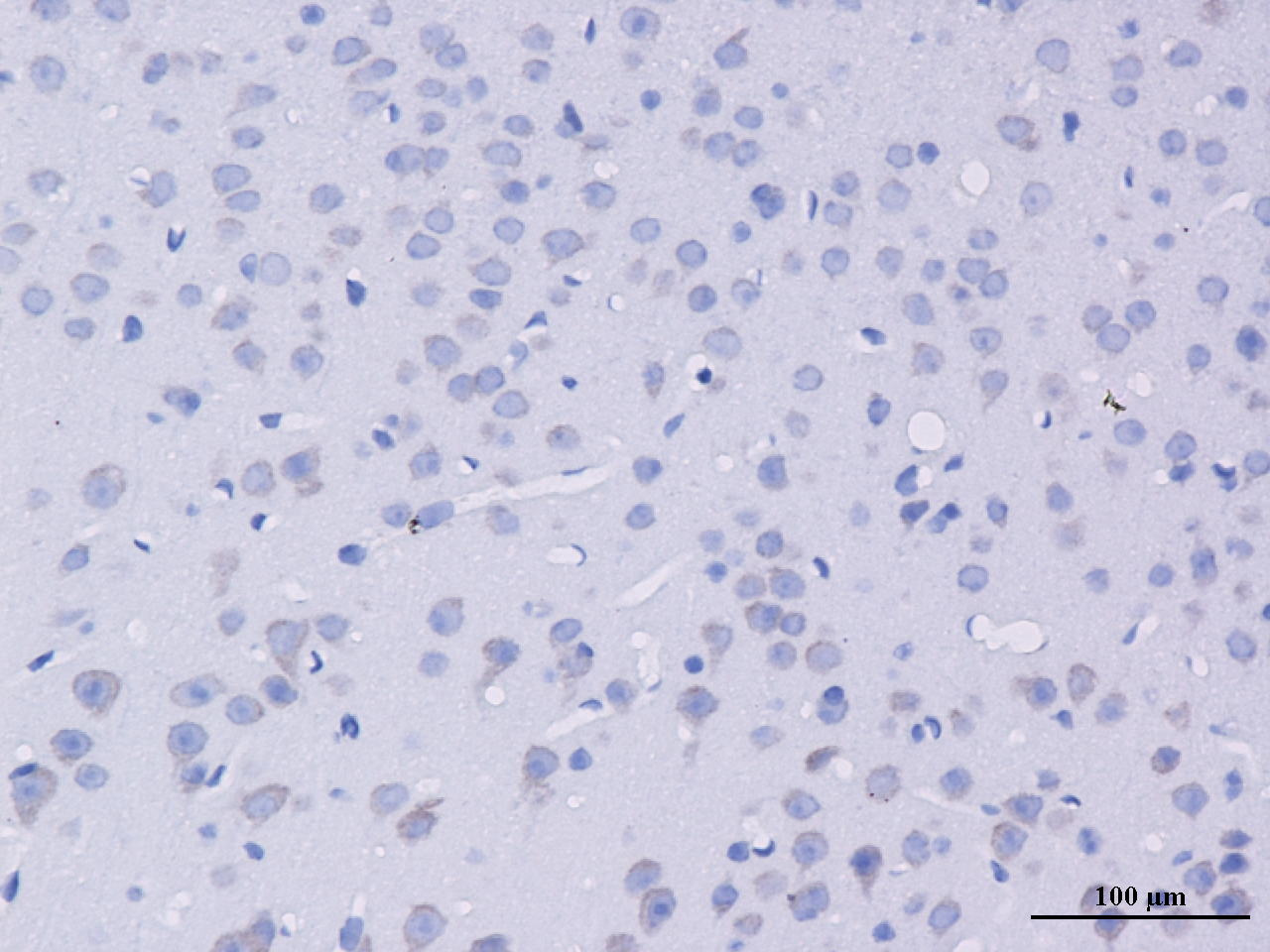

Supplement: Supplementary file 4 [file Data_Sheet_4.ZIP › Immunohistochemistry-KHK/22.Figure 5E. zokor-KHK-21%.tif]

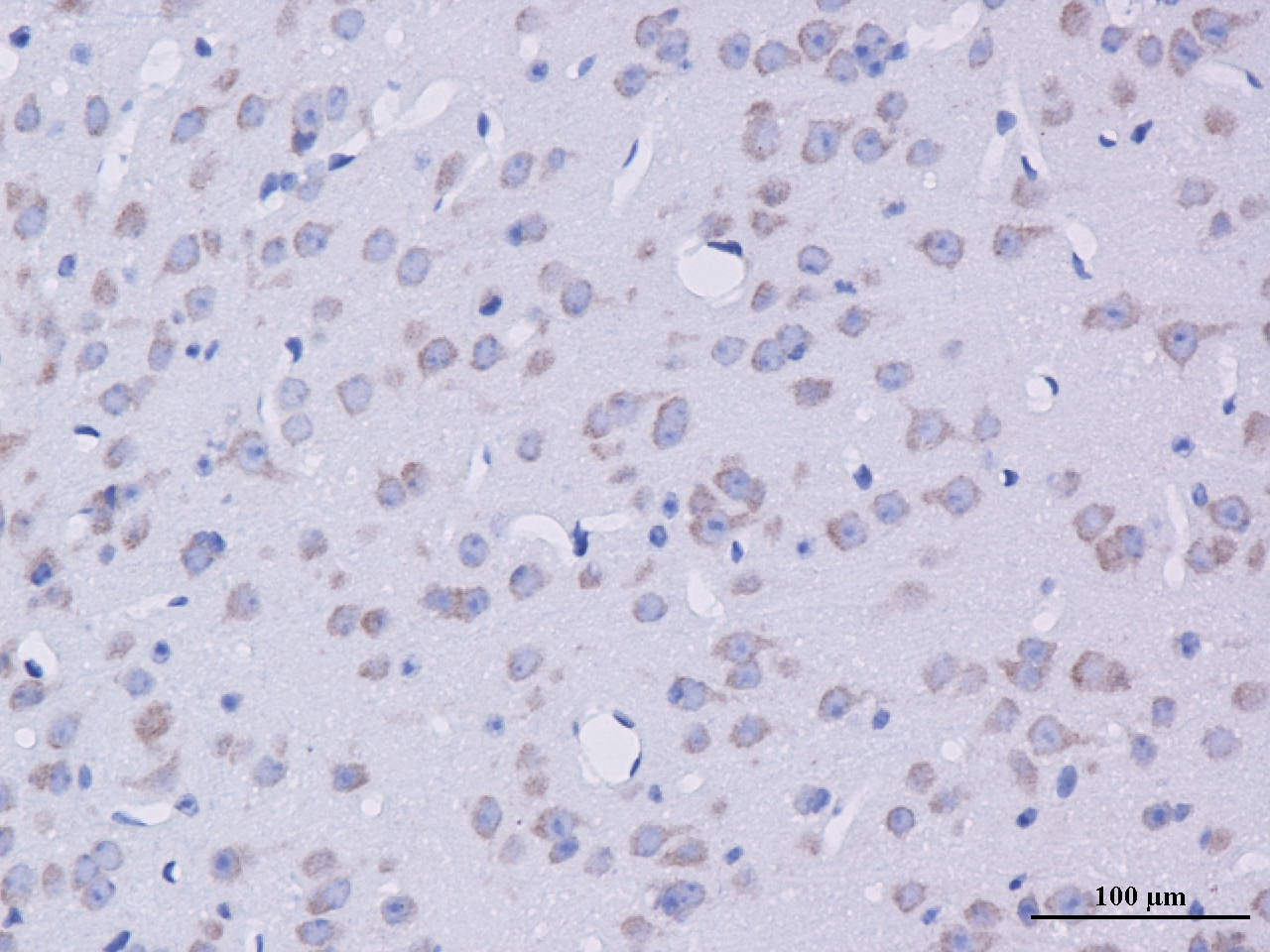

Supplement: Supplementary file 4 [file Data_Sheet_4.ZIP › Immunohistochemistry-KHK/23.Figure 5E. zokor-KHK-10.5%.tif]

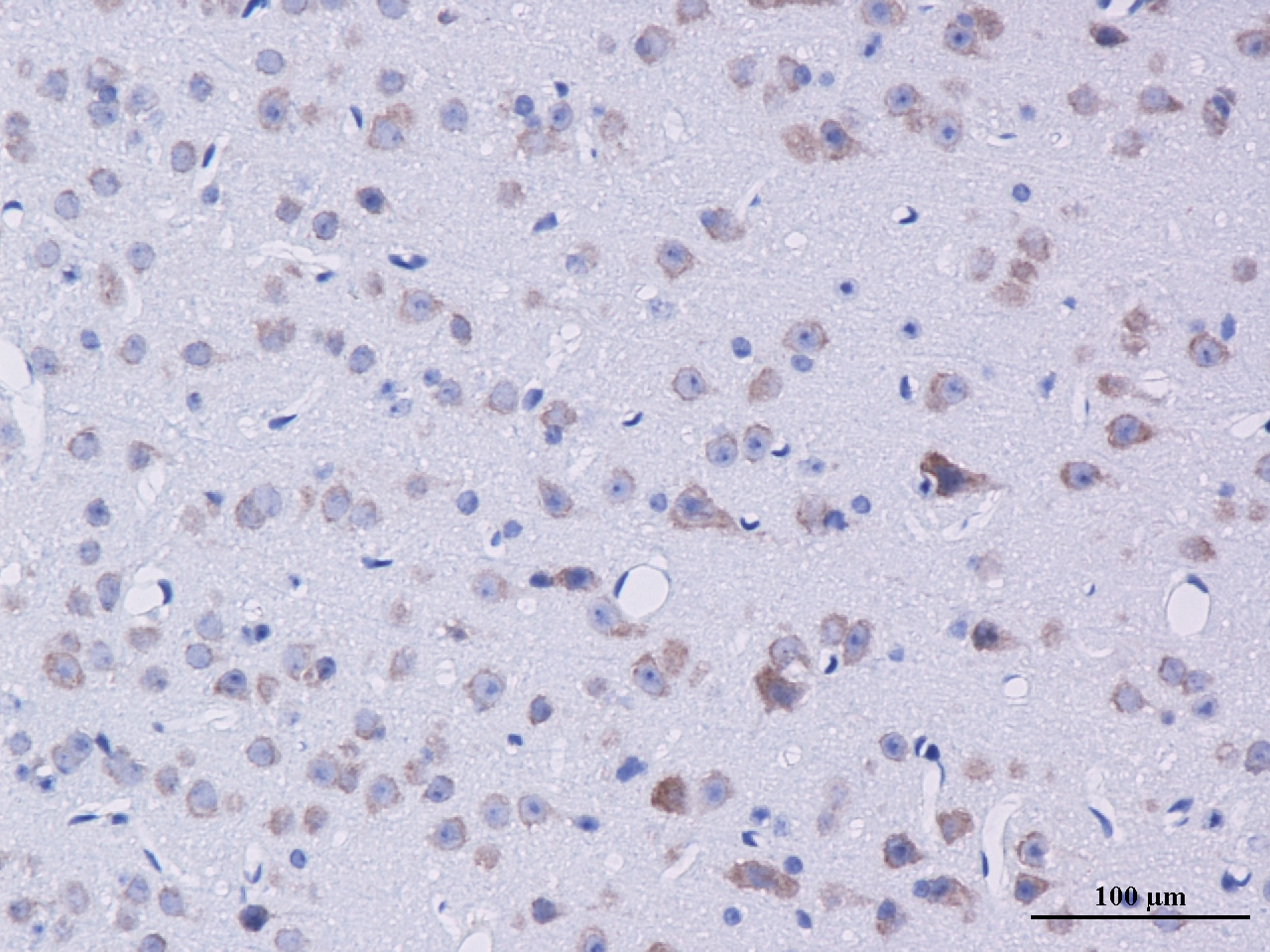

Supplement: Supplementary file 4 [file Data_Sheet_4.ZIP › Immunohistochemistry-KHK/24Figure 5E. zokor-KHK-6.5%.tif]

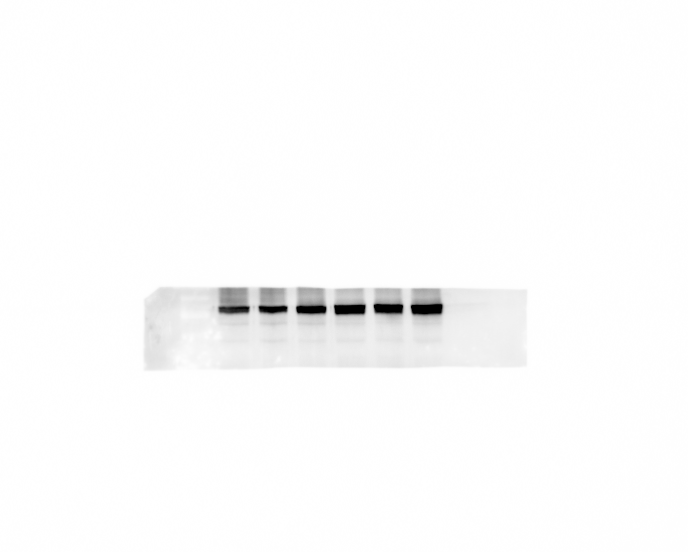

Supplement: Supplementary file 5 [file Data_Sheet_5.ZIP › Western blot original image/Figure.4-A GLUT1.tif]

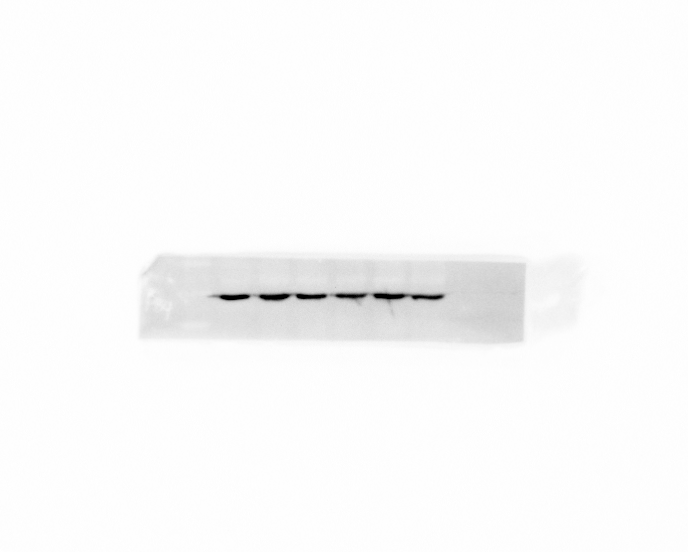

Supplement: Supplementary file 5 [file Data_Sheet_5.ZIP › Western blot original image/Figure.4-A a┬-actin.tif]

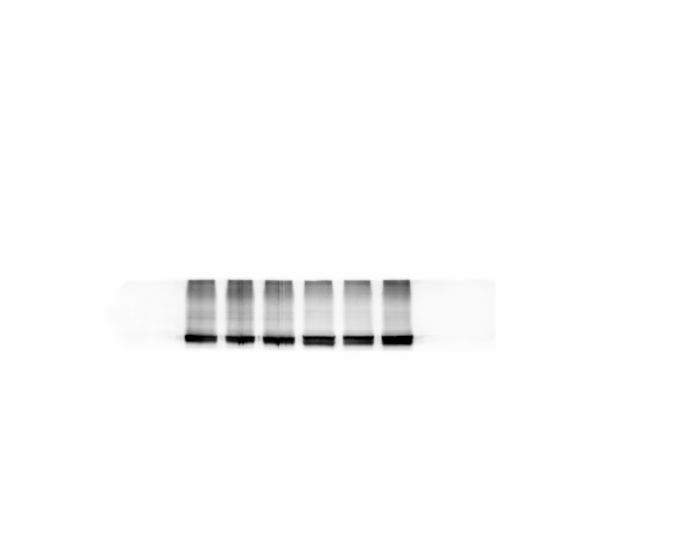

Supplement: Supplementary file 5 [file Data_Sheet_5.ZIP › Western blot original image/Figure.4-B PFK.tif]

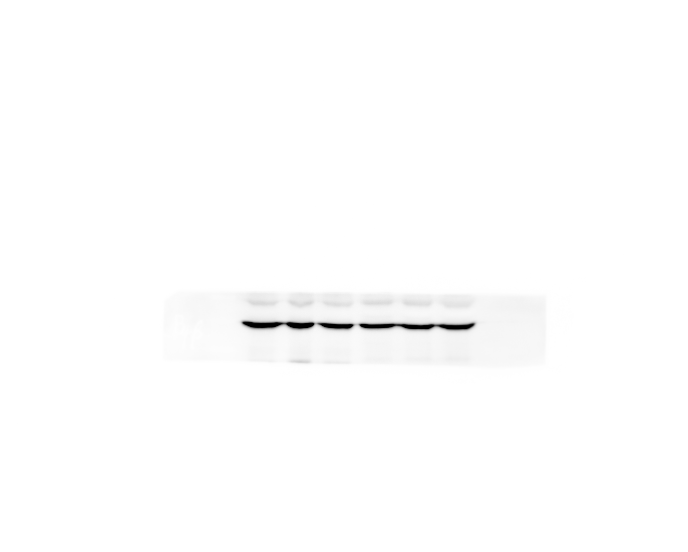

Supplement: Supplementary file 5 [file Data_Sheet_5.ZIP › Western blot original image/Figure.4-B a┬-actin.tif]

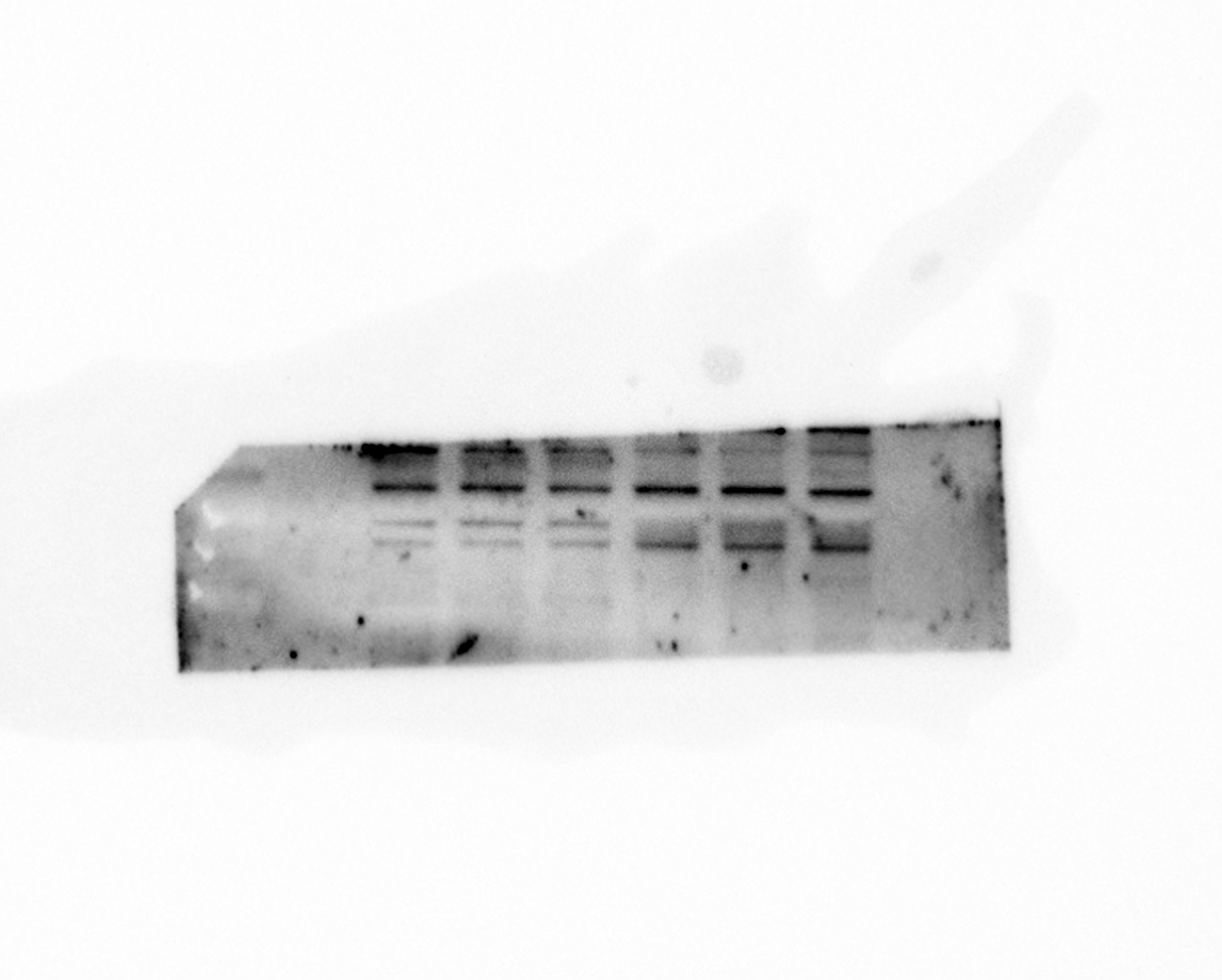

Supplement: Supplementary file 5 [file Data_Sheet_5.ZIP › Western blot original image/Figure.4-C GLUT5.Tif]

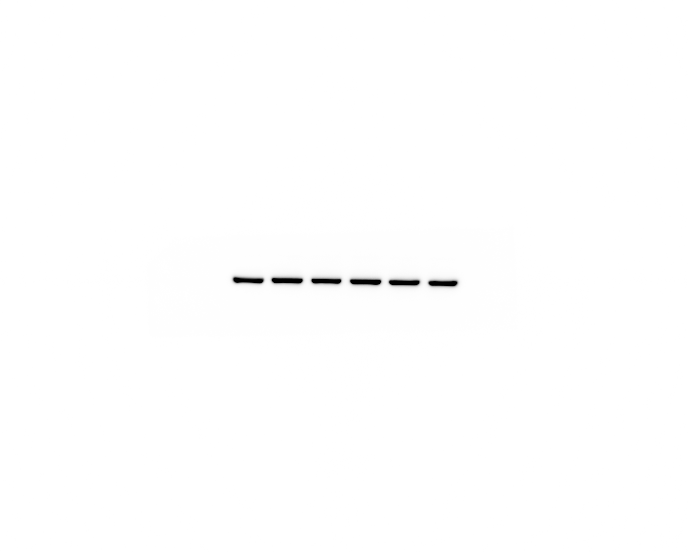

Supplement: Supplementary file 5 [file Data_Sheet_5.ZIP › Western blot original image/Figure.4-C a┬-actin.tif]

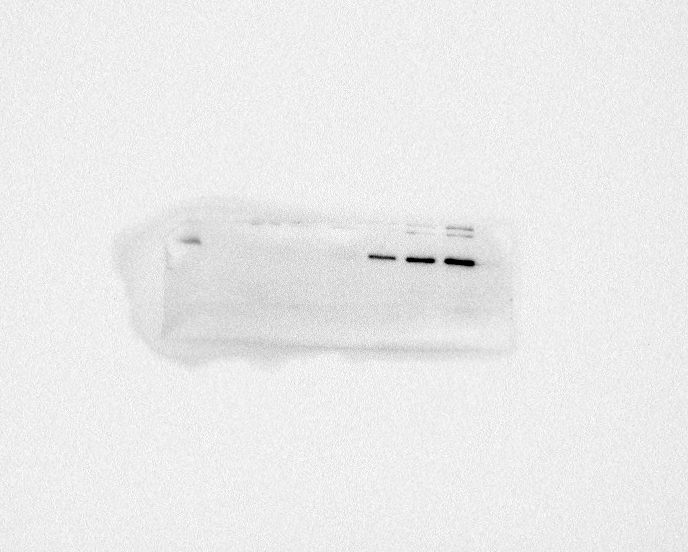

Supplement: Supplementary file 5 [file Data_Sheet_5.ZIP › Western blot original image/Figure.4-D KHK.tif]

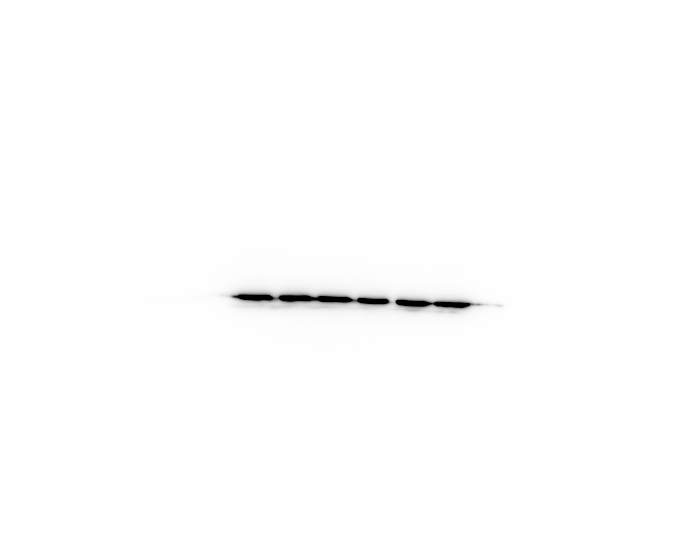

Supplement: Supplementary file 5 [file Data_Sheet_5.ZIP › Western blot original image/Figure.4-D a┬-actin.tif]
